# Distinct CTC Specific RNA Profile Enables NSCLC Early Detection and Dynamic Monitoring of Advanced NSCLC

**DOI:** 10.1002/advs.202417849

**Published:** 2025-04-03

**Authors:** Xiaoyu Wang, Pi Ding, Wenjuan Xu, Lei Qiu, Jing Ren, Yucheng Fei, Zhili Wang, Cheng Li, Yufei Xing, Mingjing Shen, Yawen Zhu, Yun Guo, Na Sun, Renjun Pei, Minhua Shi

**Affiliations:** ^1^ Department of Pulmonary and Critical Care Medicine The Second Affiliated Hospital of Soochow University Suzhou 215004 China; ^2^ CAS Key Laboratory for Nano‐Bio Interface Suzhou Institute of Nano‐Tech and Nano‐Bionics Chinese Academy of Sciences Suzhou 215123 China; ^3^ School of Nano‐Tech and Nano‐Bionics University of Science and Technology of China Hefei 230026 China; ^4^ Department of Gynecology and Obstetrics The Second Affiliated Hospital of Soochow University Suzhou 215004 China; ^5^ Thoracic Department The Second Affiliated Hospital of Soochow University Suzhou 215004 China

**Keywords:** circulating tumor cells, dynamic monitor, liquid biopsy, mRNA, NSCLC

## Abstract

Circulating tumor cells (CTCs) hold significant potential as biomarkers for the diagnosis and management of non‐small cell lung cancer (NSCLC). However, their clinical utility is limited by the heterogeneity of CTC subtypes and the need for robust, quantitative assays. In this study, a quantitative CTC RNA assay incorporating multi‐antibody‐based CTC isolation and specific mRNA quantification by RT‐ddPCR is developed. Two distinct models are established: NSCLC CTC Score^D^ for detecting early NSCLC (stages I‐II), and NSCLC CTC Score^M^ for monitoring advanced NSCLC (stages III‐IV), based on distinct cohort criteria. NSCLC CTC Score^D^ demonstrates high diagnostic performance for early‐stage NSCLC, achieving an area under the receiver operating characteristic curve (AUC) of 0.93, significantly outperforming serum CEA (AUC = 0.70). Compared to NSCLC CTC Score^D^, NSCLC CTC Score^M^ captures a key gene feature of *KRT19*, whose fragment protein, serum CYFRA 21‐1, is used as a prognostic biomarker for advanced NSCLC. Notably, CTC Scores^M^ exhibits a more accurate early warning of patient responses to different therapies than serum CYFRA21‐1 levels, which may provide a potential blood test‐based biomarker for improved treatment assessment in advanced NSCLC.

## Graphical abstract

A quantitative CTC RNA assay is developed by incorporating multi‐antibody‐based CTC isolation and specific mRNA quantification using RT‐ddPCR. The NSCLC CTC Score^D^ demonstrates high accuracy for early‐stage NSCLC detection, significantly outperforming serum CEA. NSCLC CTC Score^M^ exhibits a more accurate early‐warning of responses to different therapies than serum CYFRA21‐1 levels, providing a potential blood test‐based biomarker for advanced NSCLC management.

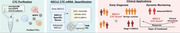

## 1 Introduction

According to “Cancer Statistics 2024”, lung cancer remains the first leading cause of cancer deaths worldwide.^[^ ^1^ ^]^ In China, the number of lung cancer deaths is as high as 733 000, accounting for 23.8% of all cancer deaths in 2022.^[^ ^2^ ^]^ The 5‐year survival rate is quite low, largely due to late‐stage diagnosis and limited efficacy of treatments for advanced disease.^[^ ^3^ ^]^ In recent years, the development of imaging techniques, such as low‐dose computed tomography (LDCT), have improved the early detection rate of pulmonary nodules, particularly for non‐small cell lung cancer (NSCLC).^[^ ^4^, ^5^ ^]^ However, there are still some potential harms associated with image screening, such as false‐positive results, which can lead to unnecessary patient anxiety, invasive procedures, and additional healthcare costs.^[^ ^6^, ^7^, ^8^ ^]^ Therefore, there is an urgent need to develop a method that can accurately detect NSCLC at early stage, reliably differentiating malignant nodules from benign nodules. On the other hand, for patients with advanced NSCLC, current treatments such as chemotherapy, immunotherapy, and targeted therapies often face challenges, including variable patient response rates and the emergence of drug resistance.^[^ ^9^, ^10^ ^]^ Real‐time monitoring of NSCLC during treatment is essential for assessing therapeutic efficacy and detecting resistance early. However, effective clinical methods for such monitoring are limited.

Circulating tumor cells (CTCs) offer a promising solution to address both diagnostic and monitoring challenges in NSCLC,^[^ ^11^, ^12^ ^]^ which are tumor‐derived cells that shed into the blood circulation, and enable minimally invasive “liquid biopsies” that can be sampled repeatedly to provide real‐time insights into tumor progression and treatment response.^[^ ^13^, ^14^, ^15^ ^]^ While CTCs are recognized as potential biomarkers for NSCLC, their clinical application faces several challenges, such as difficulties in standardizing sample preparation and isolation method, and CTC heterogeneity in surface marker expression.^[^ ^16^, ^17^ ^]^ It is well known that CTCs are generated through epithelial‐to‐mesenchymal transition (EMT) in epithelial cancers, which reduces the EpCAM expression on NSCLC CTCs,^[^ ^18^, ^19^, ^20^ ^]^ limiting the effectiveness of traditional EpCAM‐based enrichment methods. Moreover, distinguishing between tumor‐derived CTCs and normal circulating epithelial cells remains difficult, potentially leading to false positives and lowering diagnostic specificity.^[^ ^21^, ^22^, ^23^ ^]^ To solve these issues, many researchers tried to use a broader range of markers to target and capture a more comprehensive CTC profile.^[^ ^24^, ^25^, ^26^ ^]^ Furthermore, molecular analysis of these diverse CTC populations also enables a more specific and comprehensive diagnostic approach for NSCLC.^[^ ^27^, ^28^ ^]^

**Figure 1:**
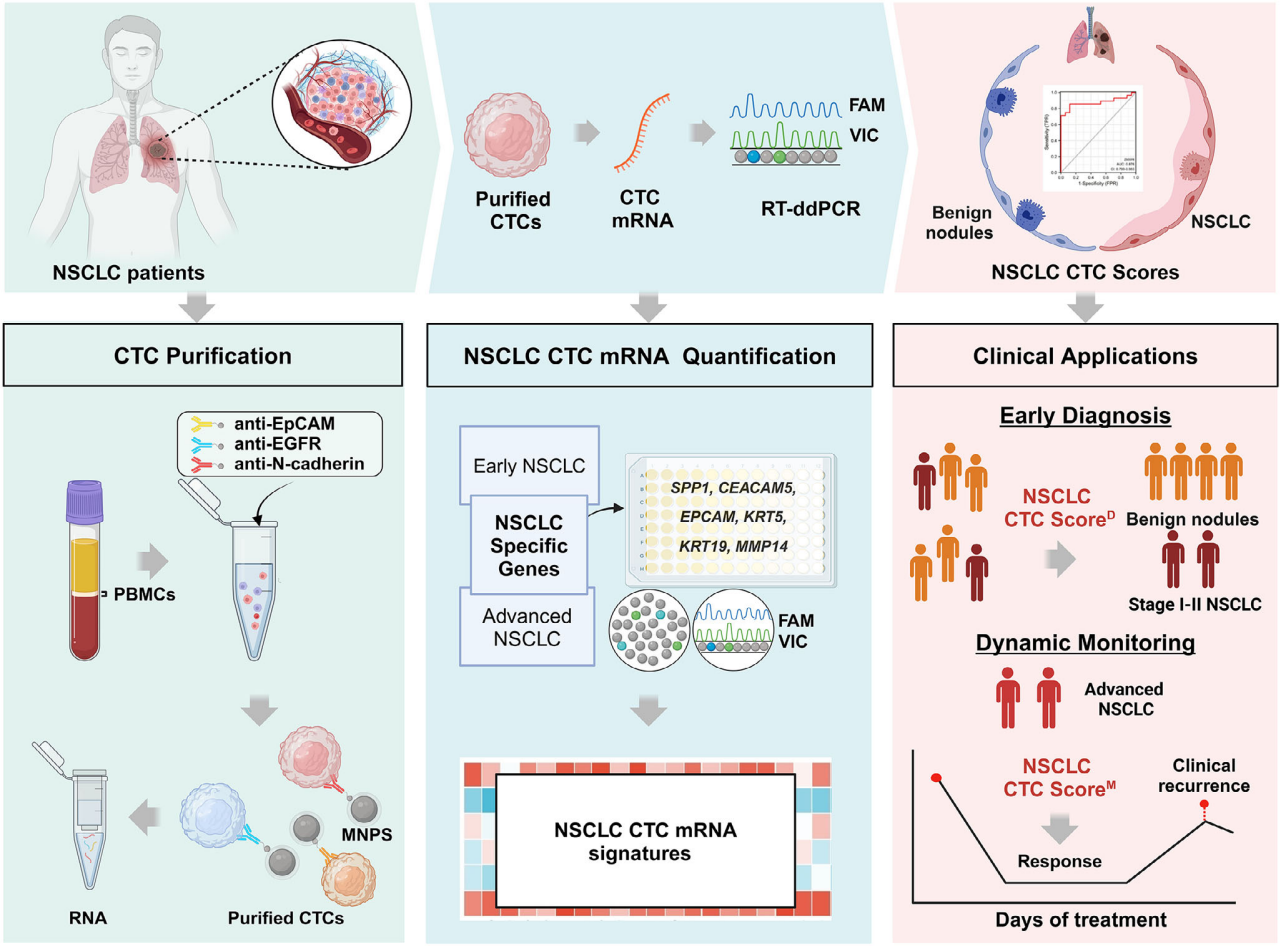
The workflow of developing a quantitative CTC RNA assay for NSCLC clinical sample test in this study.

In this study, we developed a quantitative CTC RNA assay to explore the diagnostic potential of CTCs in NSCLC, as illustrated in **Figure** **1**. To address the inherent heterogeneity of CTCs in NSCLC, we employed a combination of antibodies targeting EpCAM, EGFR, and N‐cadherin for CTC isolation. This approach enabled the capture of a broader spectrum of CTC subtypes. Subsequently, a panel of NSCLC‐specific genes was utilized to quantify tumor‐associated mRNA within the isolated CTCs, facilitating the assessment of tumor‐specific gene expression profiles. CTC mRNA was quantified using reverse transcription digital droplet PCR (RT‐ddPCR). As RNA profiles in CTCs differ significantly between early and advanced stages, we developed two distinct models, NSCLC CTC Score^D^ for detecting early‐stage NSCLC (stages I‐II) and NSCLC CTC Score^M^ for dynamically monitoring advanced‐stage NSCLC (stages III‐IV), with well‐defined cohort criteria, respectively. The distinct molecular profiles identified by NSCLC CTC Score^D^ and NSCLC CTC Score^M^ underscore their complementary roles in early detection and advanced disease management, respectively.

## 2 Results and Discussion

### 2.1 Screening of NSCLC Characteristic mRNA Genes

**Figure 2:**
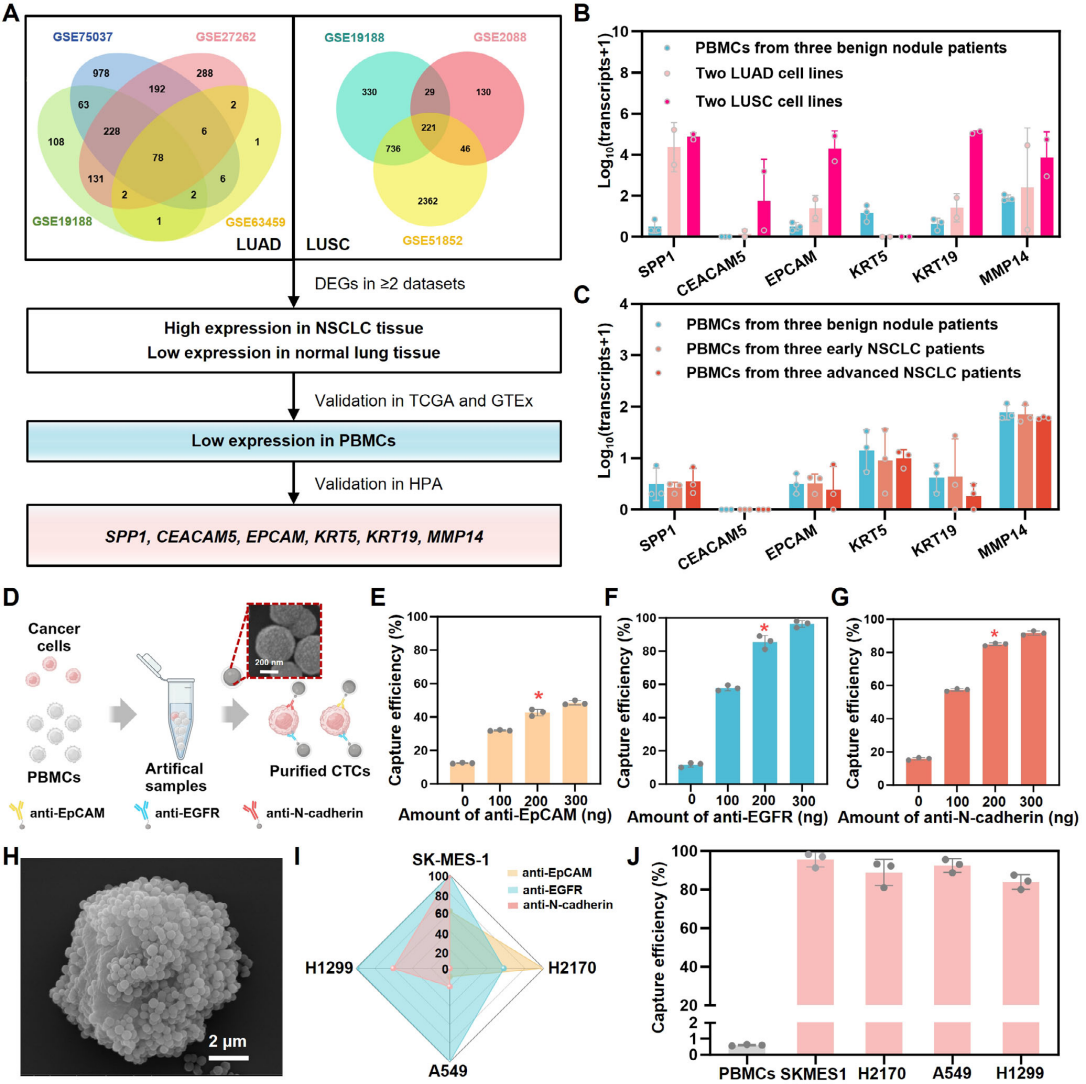
NSCLC characteristic mRNA selection and optimization of CTC purification. A) Illustration of NSCLC specific mRNA selection. B) Expression levels of 6 genes in PBMCs and four NSCLC cell lines. C) Expression levels of 6 genes in PBMCs isolated from different patients. D) Illustration of CTC purification using artificial samples. Optimization of concentrations of E) anti‐EpCAM, F) anti‐EGFR, and G) anti‐N‐cadherin for CTC capture. H) A respective SEM image of an isolated cancer cell with mounts of MNPs on its surface. I) The relative expression levels of EpCAM, EGFR, and N‐cadherin on these four cell lines were analyzed by flow cytometry. J) Capture performance of the antibody cocktail‐modified MNPs for the four cancer cell lines.

Data from multiple Gene Expression Omnibus (GEO) datasets were analyzed to identify differentially expressed genes (DEGs) between NSCLC and normal lung tissues. For lung adenocarcinoma (LUAD), datasets GSE19188, GSE75037, GSE27262, and GSE63459 were included, and for lung squamous cell carcinoma (LUSC), datasets GSE19188, GSE2088, and GSE51852 were analyzed, as illustrated in **Figure** **2**A. Candidate genes were selected based on high expression in NSCLC tissues, and low expression in normal lung tissues. Then these genes were further validated in public datasets involving NSCLC tissues (from TCGA) and normal lung tissues (from GTEx). Low expression levels in peripheral blood mononuclear cells (PBMCs) were also confirmed in public data from the Human Protein Atlas (HPA). This approach identified six potential NSCLC‐specific markers for NSCLC CTC analysis, secreted phosphoprotein 1 (*SPP1*), carcinoembryonic antigen‐related cell adhesion molecule 5 (*CEACAM5*), epithelial cell adhesion molecule (*EPCAM*), keratin 5 (*KRT5*), keratin 19 (*KRT19*), and matrix metallopeptidase 14 (*MMP14*). Their expression levels in NSCLC tissues (from TCGA) and normal lung tissues (from GTEx) are shown in Figure S1 (Supporting Information). To elucidate the biological functions of our 6‐gene panel, we conducted pathway analysis and enriched the terminology related to various functional categories, including biological processes (BP), molecular functions (MF), and cellular components (CC), as illustrated in Figure S2 (Supporting Information).

To confirm the specificity of these six genes, we examined their expression levels in PBMCs isolated from whole blood samples of three patients with benign lung nodules and four NSCLC cell lines, including two LUAD cell lines (A549 and H1299), and two LUSC cell lines (SK‐MES‐1 and H2170). The results, summarized in Figure 2B, revealed low expression levels of all six genes in PBMCs. In contrast, *SPP1* and *MMP14* were highly expressed in both LUAD and LUSC cell lines. Meanwhile, *CEACAM5*, *EPCAM*, and *KRT19* exhibited high expression levels specifically in LUSC cell lines.

### 2.2 Purification of NSCLC CTCs

Prior to CTC purification, we conducted a small pilot test to directly quantify the mRNA expression levels of six genes in PBMC samples. Samples were obtained from three patients with benign lung nodules, three patients with early‐stage NSCLC (stage I‐II), and three patients with advanced NSCLC (stage III‐IV). Low copy numbers of all six mRNAs were detected across all groups, with no significant difference observed between benign disease and NSCLC (Figure 2C). The results underscore the critical importance of CTC purification to eliminate background noise from PBMCs and enable more specific molecular analysis of tumor‐derived signals.

To capture the heterogeneity of NSCLC CTC characteristics, magnetic nanoparticles (MNPs) functionalized with an antibody cocktail targeting EMT‐related surface markers (EpCAM, EGFR, and N‐cadherin) were used for CTC isolation. Artificial samples were prepared by spiking 200 SK‐MES‐1 cells into a solution containing 2×10^6^ PBMCs (in 200 µL solution) to optimize the concentrations of the three antibodies for cell capture, as illustrated in Figure 2D. The results in Figure 2E–G demonstrated that the optimal concentrations of anti‐EpCAM, anti‐EGFR, and anti‐N‐cadherin were 200, 200, and 200 ng, respectively, for efficient CTC capture from a 200 µL solution derived from 2 mL whole blood samples. Figure 2H and Figure S3 (Supporting Information) show scanning electron microscopy (SEM) images of isolated cancer cells with MNPs embedded on the surface. Obvious aggregation of these nanoparticles on the cell surface can be observed. To confirm the exist of MNPs on the captured cells, we characterized the distribution of Fe element using energy dispersive X‐ray spectroscopy (EDS). Then, two LUAD cell lines (A549 and H1299), and two LUSC cell lines (SK‐MES‐1 and H2170) were utilized for CTC purification studies using the antibody cocktail at their optimized concentrations. The expression levels of EpCAM, EGFR, and N‐cadherin on these four cell lines were analyzed by flow cytometry (Figure 2I; Figure S4, Supporting Information), high expression levels of EpCAM on H2170 cells and N‐cadherin on SK‐MES‐1 cells were observed, revealing distinct cell subtypes that mimic EMT‐like states with significant heterogeneity. Moreover, all four cell lines exhibited high expression of EGFR. The capture performance of the antibody cocktail‐modified MNPs for the four cancer cell lines was evaluated, yielding capture efficiencies ranging from 80.0% to 98.2%, as shown in Figure 2J.

### 2.3 Clinical Cohort Study

**Figure 3:**
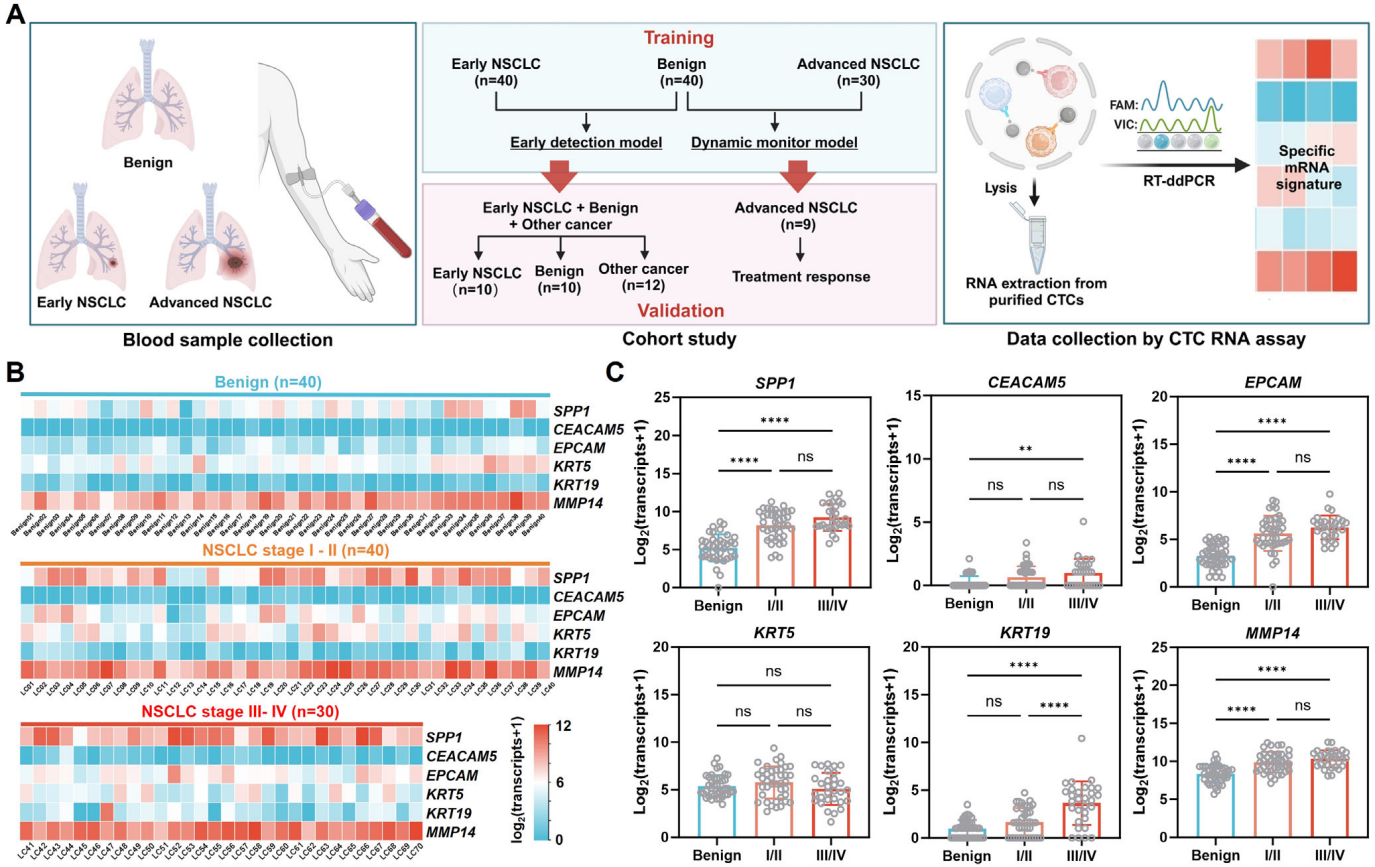
Clinical cohort study. A) llustration of the cohort study for the clinical validation of the NSCLC CTC RNA assay. B) Heatmap and C) Boxplot of NSCLC CTC‐derived six mRNA signatures of individuals in training cohort.

To evaluate the potential applications of the NSCLC CTC RNA assay, we conducted a training/validation cohort study using clinical samples, as illustrated in **Figure** **3**A. Current CTC related clinical study cohorts often lack standardized inclusion criteria for early‐ and advanced‐stage cancer patients. Notably, the RNA characteristics of CTCs vary from early to advanced stages. Consequently, applying early diagnostic models to advanced stage patients may fail to capture key molecular features, potentially leading to the loss of critical information needed for effective patient monitoring. To address this, our study aimed to develop and validate two different models with well‐defined cohort criteria, respectively: one for detecting early‐stage NSCLC (stages I‐II) and another for dynamically monitoring advanced‐stage NSCLC (stages III‐IV).

For the early detection model development, the training cohorts included patients with benign nodules (*n* = 40) and treatment‐naive early‐stage NSCLC patients (stage I‐II, *n* = 40). An independent validation cohort was also established, including patients with benign nodules (*n* = 10), patients with other cancers (*n* = 12), treatment‐naive early‐stage NSCLC patients (stage I‐II, *n* = 10). For the advanced NSCLC monitoring model, patients with benign nodules (*n* = 40) and treatment‐naive advanced NSCLC patients (stage III‐IV, *n* = 30) were included in the training cohort. Blood samples were also collected for patients during treatment to validate the dynamic monitoring performance of disease progression. Clinical characteristics of all patients are summarized in Tables S2–S5 (Supporting Information). Blood samples (2 mL each) were processed for NSCLC CTC purification following PBMC isolation, and the six mRNA profiles for each sample were obtained using RT‐ddPCR. The reproducibility of our assay was then confirmed using triplicate blood samples from three NSCLC I/II patients, and the results are summarized in Figure S5 (Supporting Information).

### 2.4 Generation and Validation of NSCLC CTC Score^D^ for Early NSCLC Detection

**Figure 4:**
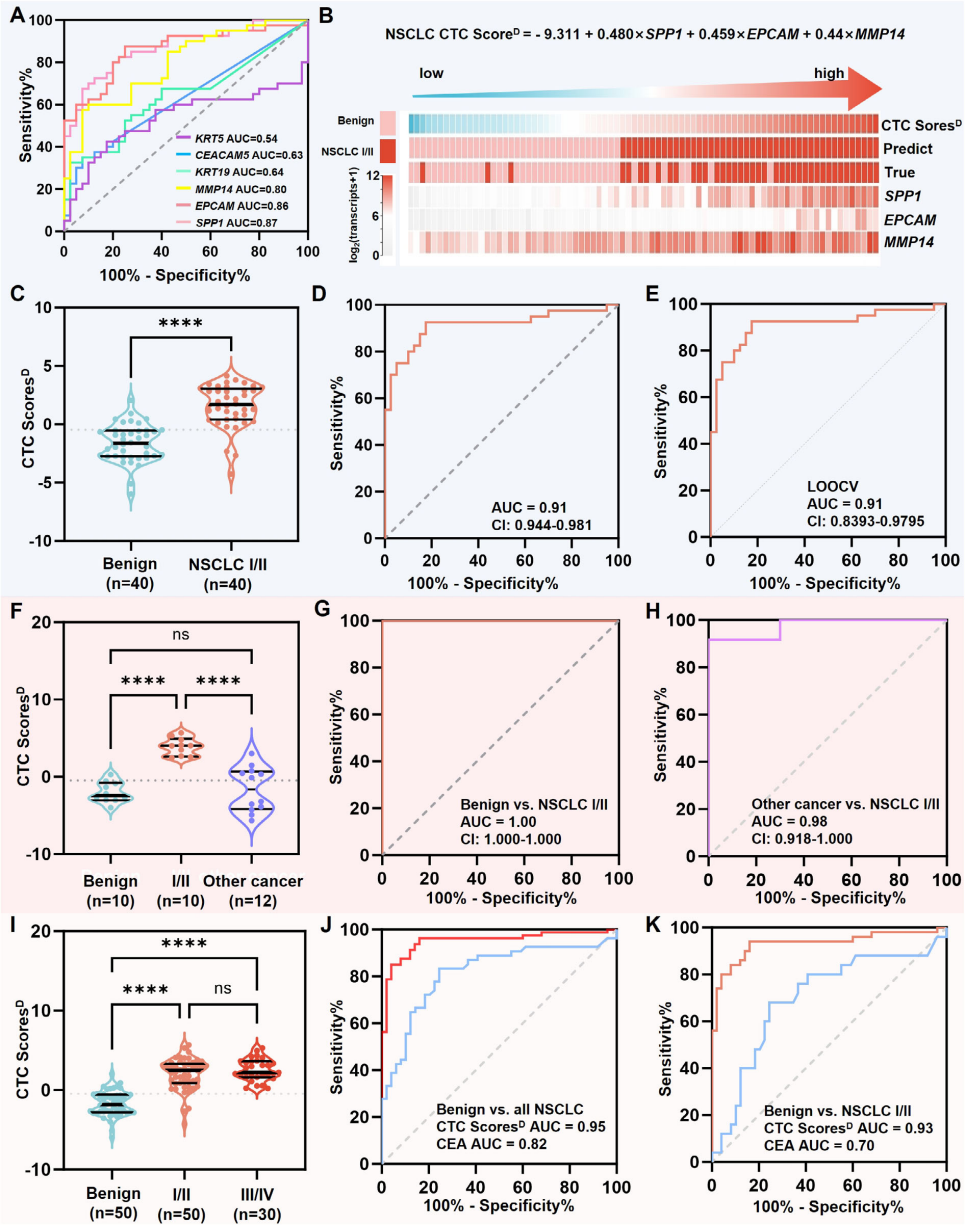
Generation and validation of NSCLC CTC Score^D^ for early NSCLC detection. A) Receiver operating characteristic curve analysis of individual gene in differentiating early‐stage NSCLC from benign nodules. B) The visualization of CTC Score^D^ for each individual in training cohort. C) A violin plot of CTC Scores^D^ in early‐stage NSCLC group and benign nodule group. D) Receiver operating characteristic curve analysis and E) ROC curve after LOOCV analysis on the training cohort. F) A violin plot of CTC Scores^D^ for individuals in an independent validation cohort. Receiver operating characteristic curve analysis of CTC Scores^D^ for differentiating early‐stage NSCLC from G) benign nodules and H) other cancers. I) A violin plot of CTC Scores^D^ in benign controls, early‐stage NSCLC patients (stage I‐II), and advanced NSCLC (stage III‐IV) across whole cohort. Receiver operating characteristic curve analysis of NSCLC CTC Score^D^ and serum CEA differentiating benign controls form J) all NSCLC patients and K) early‐stage NSCLC patients.

For NSCLC early detection study, a training cohort includes 40 treatment‐naive stage I‐II NSCLC patients and 40 patients with benign pulmonary nodules. The NSCLC CTC‐derived six mRNA signatures of these individuals are summarized in Figure 3B. The expression levels of *SPP1*, *CEACAM5*, *EPCAM*, *KRT19*, and *MMP14* were significantly elevated in early‐stage NSCLC patients compared to patients with benign nodules, while no significant difference was observed for *KRT5* (Figure 3C). To assess the diagnostic performance of individual genes in differentiating early‐stage NSCLC from benign nodules, receiver operating characteristic (ROC) curve analysis were performed (**Figure** **4**A). The detailed parameters of the ROC curve analysis for each gene are provided in Table S6 (Supporting Information). Based on multivariate logistic regression analysis, the expression levels of *SPP1*, *EPCAM*, and *MMP14* were selected to construct the NSCLC CTC Score^D^, defined by the following equation (Equation (1)):

(1) $$\begin{array}{ccc} NSCLC\;CTC\;Score^{D} & = & -9.311+0.480\times SPP1\\ & & +0.459\times EPCAM+0.44\times MMP14 \end{array}$$

The CTC Score^D^ for each individual in the training cohort was calculated and visualized in Figure 4B. A violin plot in Figure 4C depicts significant higher CTC Scores^D^ in early‐stage NSCLC group compared to the benign nodule group. The NSCLC CTC Score^D^ exhibited excellent performance in differentiating early‐stage NSCLC from benign controls with an area under the ROC of 0.91 (95% CI: 0.944 – 0.981), as shown in Figure 4D. A diagnostic accuracy of 87.5% was achieved with sensitivity of 92.5% and specificity of 82.5%, at the cut‐off value of −0.4554 (Figure S6A, Supporting Information). The diagnostic precision of CTC Scores^D^ is also up to 84.1%. The leave‐one‐out cross‐validation (LOOCV) of the training cohort was then performed, as shown in Figure 4E and Figure S6B (Supporting Information), which confirmed the accuracy of the model with an area under ROC of 0.91, sensitivity of 87.5%, and specificity of 82.5%.

(2) $$\mathrm{Accuracy}=\frac{\mathrm{correct}\;\mathrm{diagnosed}\;\mathrm{individuals}}{\mathrm{all}\;\mathrm{individuals}}$$

(3) $$\mathrm{Precision}=\frac{\mathrm{correct}\;\mathrm{diagnosed}\;\mathrm{NSCLC}}{\mathrm{individuals}\;\mathrm{diagnosed}\;\mathrm{as}\;\mathrm{NSCLC}}$$

The reliability of the NSCLC CTC Score^D^ was then tested in a small independent validation cohort, including 10 patients with benign nodules and 10 treatment‐naive early‐stage NSCLC patients. Additionally, 12 patients with other cancers were also included to validate the specificity of the gene signatures for NSCLC. The results are shown in Figure 4F. In differentiating early‐stage NSCLC from benign nodules, the CTC Scores^D^ achieved an AUC of 1.00 (Figure 4G). For distinguishing early‐stage NSCLC from other cancers, the AUC was 0.98 (95% CI: 0.918–1.000, Figure 4H).

The diagnostic performance of the NSCLC CTC Score^D^ was further evaluated in all individuals enrolled in both training and validation cohorts (50 patients with benign nodules and 50 early‐stage NSCLC patients), as well as 30 patients with advanced NSCLC. This was compared with the serum CEA level and other serum markers, which are clinical screening biomarkers for pan cancers (Figure S7, Supporting Information). Violin plot of CTC Scores^D^ in benign controls, early‐stage NSCLC patients (stage I‐II), and advanced NSCLC (stage III‐IV) are shown in Figure 4I. The NSCLC CTC Score^D^ differentiated NSCLC patients from benign controls with an AUC of 0.95 (Figure 4J), achieving a diagnostic accuracy of 91.5%, a precision of 90.6%, a sensitivity of 96.3%, and a specificity of 84.0% at the cut‐off value of −0.4554. Notably, the NSCLC CTC Score^D^ differentiated early‐stage NSCLC patients from benign controls with an AUC of 0.93, achieving a diagnostic accuracy of 89.0%, a precision of 85.5%, a sensitivity of 94.0%, and a specificity of 84% at the cutoff value of −0.4554, outperforming serum biomarker CEA (AUC = 0.70) with a diagnostic accuracy of 66.2%, a precision of 50.0%, a sensitivity of 4.0%, and a specificity of 98.0% at the cut‐off value of 5 ng mL^−1^ (Figure 4K; Figure S8 and Table S7, Supporting Information). The limited sensitivity of serum markers (CEA, sensitivity = 4.0%; CA125, sensitivity = 4.0%; CA199, sensitivity = 5.3%;) makes them incapable of early diagnosis for NSCLC. These findings demonstrate the outstanding diagnostic performance of the NSCLC CTC Score^D^ in distinguishing early‐stage NSCLC patients from benign lung nodules, with significant potential application in early NSCLC detection.

### 2.5 Development of the NSCLC CTC Score^M^ for Advanced NSCLC Diagnosis and Management

**Figure 5:**
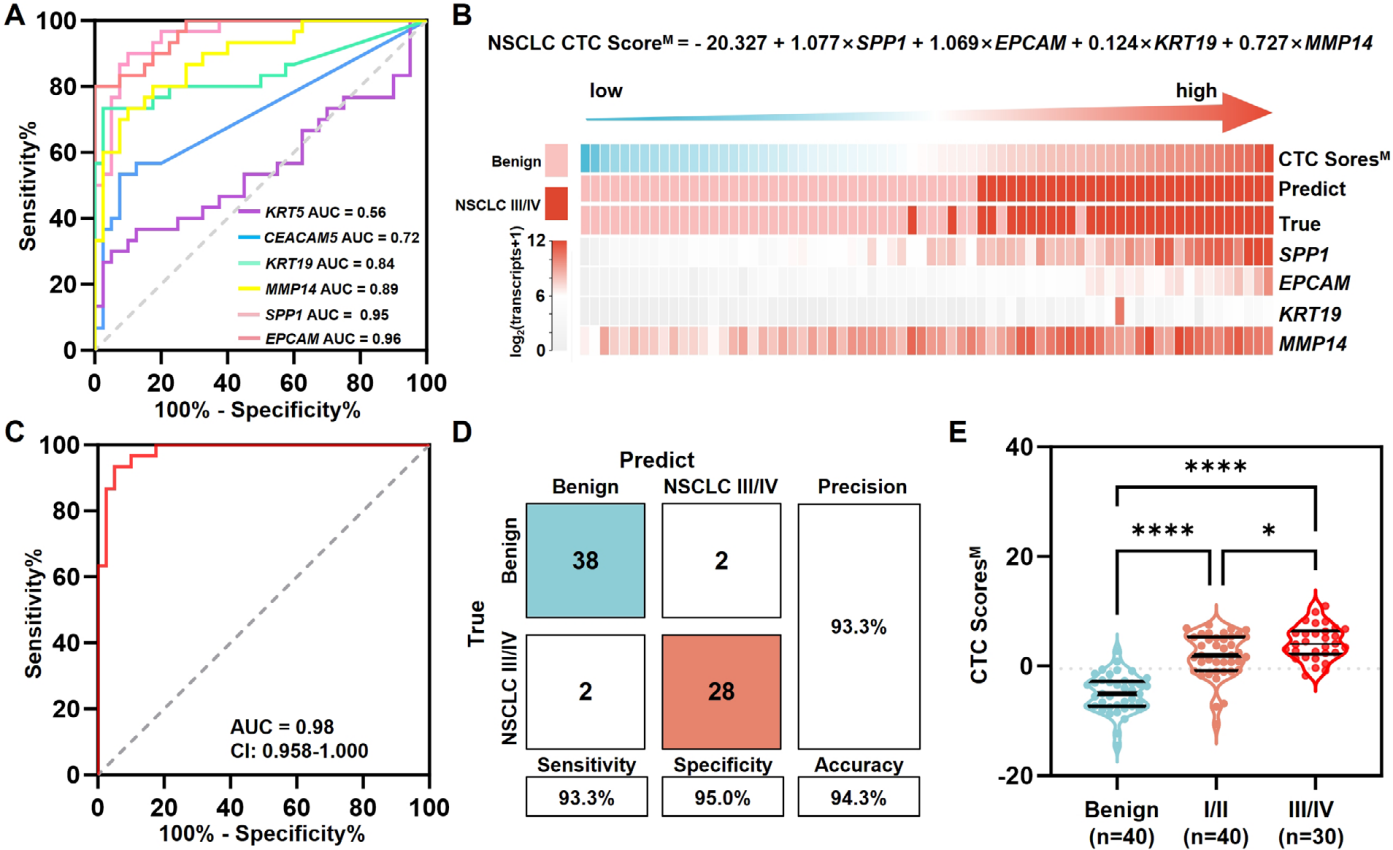
Generation of the NSCLC CTC Score^M^ for advanced NSCLC diagnosis and management. A) Receiver operating characteristic curve analysis of individual gene in differentiating advanced NSCLC from benign nodules. B) The visualization of CTC Score^M^ for each individual in training cohort. C) Receiver operating characteristic curve analysis and D) confusion matrix analysis of CTC Scores^M^ in differentiating advanced NSCLC from benign controls. E) A violin plot of CTC Scores^M^ in benign controls, early‐stage NSCLC patients (stage I‐II), and advanced NSCLC (stage III‐IV).

We noted that there was no significant difference in CTC Scores^D^ between early‐stage NSCLC group and advanced NSCLC group (Figure 4I, *p* = 0.39). In order to better capture the molecular information of CTCs in advanced NSCLC patients, a new model for advanced NSCLC diagnosis was generated. For the advanced NSCLC study, the training cohorts include 30 treatment‐naive stage III‐IV NSCLC patients and 40 patients with benign pulmonary nodules. CTC‐derived mRNA signatures of these individuals are summarized in Figure 3B. *KRT19* was found to be significantly more highly expressed in advanced NSCLC patients compared to early‐stage NSCLC patients (Figure 3C), highlighting its potential to reveal the tumor information specific to advanced disease. This finding coincides with the clinical use of serum CYFRA21‐1, which is a cytokeratin‐19 fragment, as a biomarker of recurrence or therapeutic efficacy for NSCLC. It is also noted that there is no significant difference in the expression levels in most of these genes between adenocarcinoma (*n* = 22) and squamous carcinoma (*n* = 8) (Figure S9, Supporting Information). ROC curve analysis identified *SPP1*, *EPCAM*, *MMP14*, and *KRT19* as significant markers for distinguishing advanced NSCLC from benign nodules (**Figure** **5**A). The detailed parameters of the ROC curve analysis for each gene are provided in Table S8 (Supporting Information). These genes were used to develop the NSCLC CTC Score^M^ via multivariate logistic regression and the equation is shown in Equation (4):

(4) $$\begin{array}{ccc} NSCLC\;CTC\;Score^{M} & = & -20.327+1.077\times SPP1\\ & & +1.069\times EPCAM+0.124\times KRT19\\ & & +0.727\times MMP14 \end{array}$$

Significant higher scores of advanced NSCLC patients were obtained compared to the benign nodule group (Figure 5B). It achieved an AUC of 0.98 (95% CI: 0.958‐1.000) for differentiating advanced NSCLC from benign nodules, with a diagnostic accuracy of 94.3%, sensitivity of 93.3%, specificity of 95.0%, and precision of 93.3% (Figure 5C,D). The comparation of CTC Scores^M^ for all individuals enrolled in the training cohort (*n* = 110) is summarized in Figure 5E. The NSCLC CTC Score^M^ demonstrated significantly higher scores in the advanced NSCLC group than both early‐stage NSCLC (*p* = 0.02) and benign nodule groups (*p* < 0.02). This result demonstrated that the NSCLC CTC Score^M^ incorporating *KRT19* captures additional tumor signals characteristic of advanced disease compared to the NSCLC CTC Score^D^. This feature makes the NSCLC CTC Score^M^ more potential for advanced NSCLC management, supporting its utility for personalized therapeutic efficacy monitoring.

### 2.6 Application of NSCLC CTC Score^M^ for Dynamic Therapeutic Monitoring of Advanced NSCLC Patients

**Figure 6:**
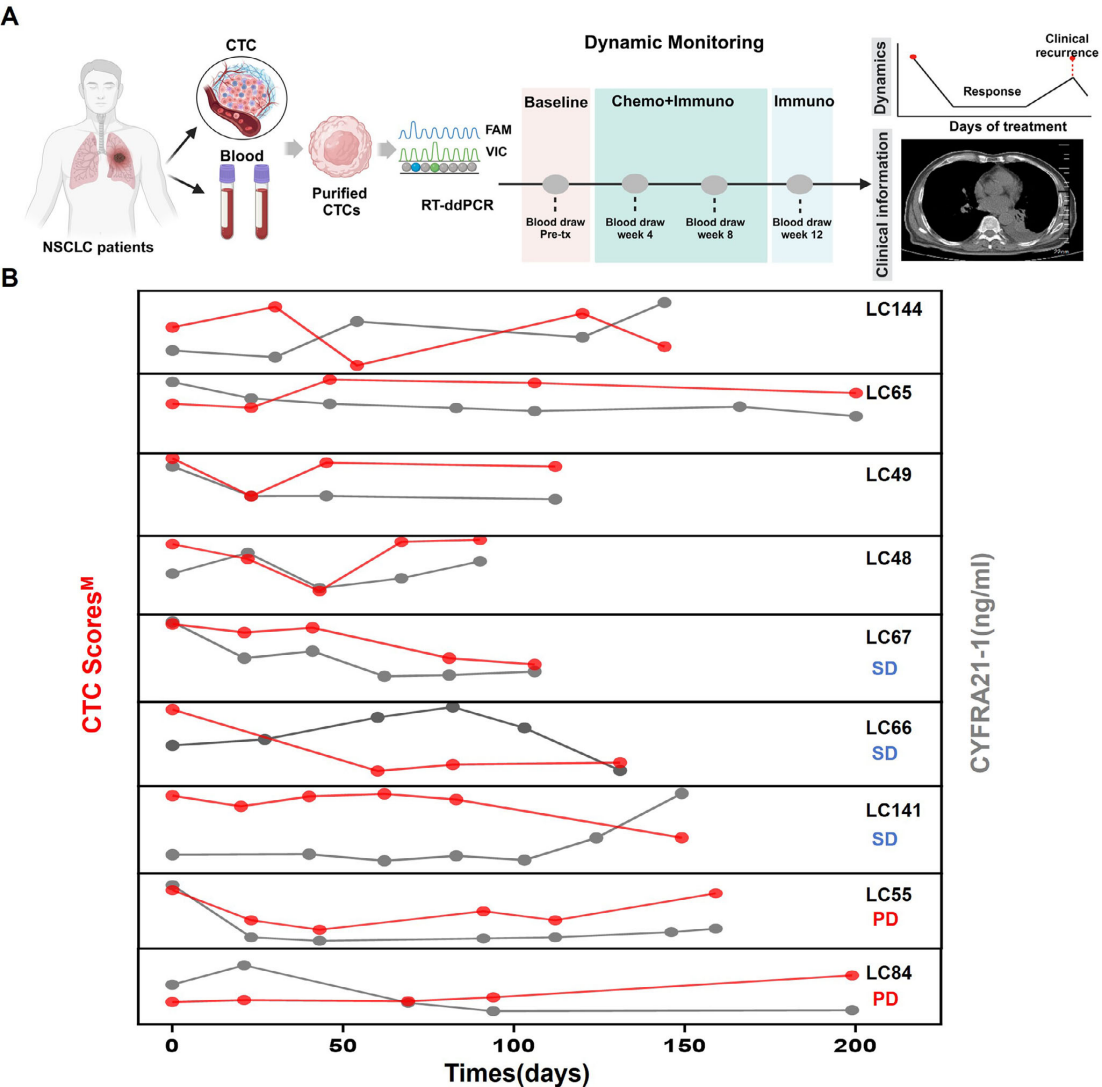
NSCLC CTC Score^M^ for dynamic therapeutic monitoring of advanced NSCLC patients. A) Illustration of NSCLC CTC Score^M^ for real‐time therapeutic monitoring in patients undergoing treatment. B) The monitoring results of CTC Scores^M^ and serum CYFRA21‐1 levels for 9 patients.

To evaluate the performance of NSCLC CTC Score^M^ for advanced NSCLC management, we longitudinally collected series of blood samples of 9 advanced patients undergoing treatment to assess their utility for real‐time therapeutic monitoring (**Figure** **6**A). Peripheral blood samples were collected from advanced NSCLC patients serially before cycles of treatment, respectively. The serum marker of CYFRA21‐1, clinically employed for advanced NSCLC monitoring, was also recorded at each timepoint of blood draw for the NSCLC CTC assay test. The monitoring results of CTC Scores^M^ and serum CYFRA21‐1 levels for all these 9 patients were summarized in Figure 6B. It was observed that CTC Scores^M^ varied with the treatment of each patient.

**Figure 7:**
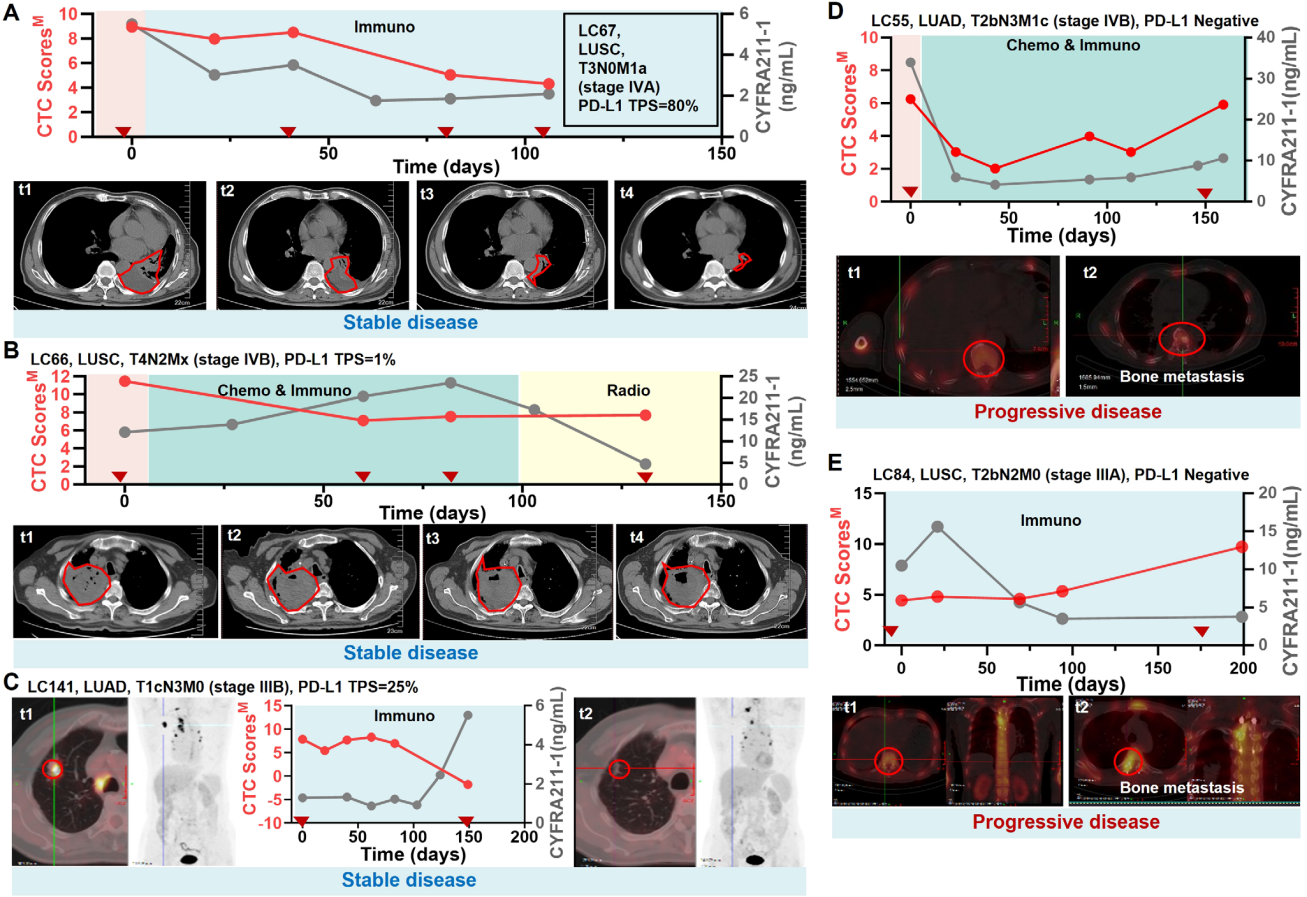
Analysis of association between CTC Scores^M^ and RECIST in dynamic monitoring of 5 advanced patients.

Using the response evaluation criteria in solid tumors (RECIST 1.1) as a primary endpoint (assessment criteria in Supporting information), we access the detailed dynamic performance of CTC Scores^M^ for 5 patients in our cohort over the course of their treatment: 3 cases of stable disease (SD, LC67, LC66, LC141) and 2 cases of progressive disease (PD, LC55, LC84). We then analyzed the association between CTC Scores^M^ and RECIST in these 5 patients, by aligning their CTC Scores^M^ with computed tomography (CT) images and serum CYFRA21‐1 levels (**Figure** **7**).

Patient LC67, a T3N0M1a stage LUSC case, was given first‐line immunotherapy with Camrelizumab with a high PD‐L1 expression (tumor proportion score, TPS = 80%). This patient demonstrated stable disease, with a continuous decline in CTC Scores^M^, which was consistent with the declined CYFRA21‐1 levels and reduced tumor burden observed on the CT images (Figure 7A).

Patient LC66, a T4N2Mx stage LUSC case, received chemo‐immunotherapy with a PD‐L1 TPS of 1%, followed by radiotherapy. A lightly enlarged cavity within the tumor was observed in CT images after the radiotherapy (Figure 7B). CTC Scores^M^ remained relatively stable, while CYFRA21‐1 levels exhibited an initial increase followed by a decline, suggesting that the two markers may offer complementary insights into tumor status.

Patient LC141, a case of recurrent LUAD (T1cN3M0 stage), received first‐line Pembrolizumab immunotherapy with a PD‐L1 TPS of 25%. Post‐treatment CT images showed reduced tumor burden, and whole‐body positron emission tomography and computed tomography (PET/CT) revealed a marked reduction in fluorine‐18‐deoxyglucose (FDG) accumulation. While serum CYFRA21‐1 levels trended upward and were not informative for tracking treatment response, CTC Scores^M^ consistently declined (Figure 7C).

Patient LC55, a T2bN3M1c stage LUAD case, had negative PD‐L1 expression and was treated with chemotherapy combined with Pembrolizumab. This patient developed multiple metastatic bone lesions. Both serum CYFRA21‐1 levels and CTC Scores^M^ indicated early warning signs of disease progression (Figure 7D).

Patient LC84, a T2bN2M0 stage LUSC case, also with PD‐L1‐negative tumor status, experienced bone metastasis after immunotherapy. Notably, serum CYFRA21‐1 levels showed a decline and failed to reflect disease progression, whereas CTC Scores^M^ demonstrated a continuous increase, offering a reliable early indicator (Figure 7E).

In summary, NSCLC CTC Score^M^ trends align closely with imaging results, such as CT and PET/CT scans, exhibiting excellent performance in monitoring the real‐time treatment response for advanced patients. More importantly, NSCLC CTC Score^M^ compensated for the limitations of serum CYFRA21‐1 levels and demonstrated consistent utility regardless of PD‐L1 status. Therefore, integrating NSCLC CTC Score^M^ with imaging‐based assessments could provide a more robust approach to improve their response evaluation.

## 3 Discussion and Conclusion

CTCs have shown great potential as a non‐invasive biomarker for cancer diagnosis, prognosis, and treatment monitoring. However, there are still several challenges that limit their full clinical translation: i) The extremely rare number and high heterogeneity of CTCs make it technically difficult to isolate them with high efficiency. ii) Standardization across different platforms and clinical studies remains a key issue, affecting the reproducibility and comparability of results. Immunoaffinity‐based CTC enrichment is a well‐known, convenient, and affordable method with potential for clinical application. To overcome the limitations of sensitivity and specificity of traditional immunoaffinity‐based CTC enrichment and to accommodate tumor heterogeneity, our assay combines two strengths: the integration of CTC capture of different phenotypes and RT‐ddPCR quantification of CTC‐derived specific mRNAs.

Since CTCs are secreted by highly heterogeneous tumors, a single capture agent may not provide sufficient capture yields, and thus missing the subpopulation of CTCs may lead to erroneous conclusions. Therefore, there is a need to develop a multi‐antibody cocktail to identify and capture CTCs in clinical samples to enable sensitive and specific detection of tumor‐derived CTCs at all stages of NSCLC. At the cellular level of analysis, we optimized the assay using simulated artificial blood samples, and the results showed that capture efficiencies as high as 80.0% to 98.2% were achieved using a multi‐antibody‐modified magnetic bead approach.

Furthermore, we developed a CTC RNA assay to quantify the NSCLC‐specific mRNA in purified CTCs, and assessed its value in NSCLC early detection and advanced patients’ therapy management. The early‐stage NSCLC detection model, NSCLC CTC Score^D^, was generated by a multivariate logistic regression method, including the expression levels of *SPP1*, *EPCAM*, and *MMP14*. No significant difference in CTC Scores^D^ was observed between the early‐stage NSCLC group and the advanced NSCLC group, which gives us a clue that the NSCLC CTC Score^D^ generated through a clearly defined cohort criteria may have a predisposition in early detection. In order to better capture the molecular information of CTCs in advanced NSCLC patients, a new model, NSCLC CTC Score^M^, for advanced NSCLC diagnosis and monitoring was generated involving *SPP1*, *EPCAM*, *MMP14* and *KRT19*. The *KRT19* mRNA has been reported as a marker of microscopic lymphatic spread for NSCLC.^[^ ^29^, ^30^ ^]^ Moreover, the serum CYFRA 21‐1 is a cytokeratin 19 (CK19) fragment and now widely used as a biomarker for NSCLC. Hence, the distinct NSCLC CTC Score^M^ can better reveal the tumor information of advanced patients and does not dependent on the expression levels of some specific genes that are highly heterogeneous across individuals.

The NSCLC CTC Score^D^ differentiated early‐stage NSCLC patients from benign controls with an AUC of 0.93, achieving a diagnostic accuracy of 89.0%, a precision of 85.5%, a sensitivity of 94.0%, and a specificity of 84%, outperforming serum biomarker CEA (AUC = 0.70), demonstrating encouraging potential in early NSCLC detection. In addition to traditional serum markers, currently recommended biomarkers in clinical include ctDNA and seven tumor‐associated autoantibodies (TAAbs) in lung cancer. As reported in a study with broad patient coverage,^[^ ^31^ ^]^ ctDNA showed a detection rate of 50% by second‐generation sequencing for stage I lung cancer with a specificity of 90%. A panel of seven TAAbs targeting antigens including p53, PGP9.5, SOX2, GAGE7, GBU4‐5, CAGE, and MAGEA1, demonstrated a sensitivity of 56.53% and a specificity of 91.60% for lung cancer diagnosis in a Chinese cohort,^[^ ^32^ ^]^ and this serum test has been used as a promising tool for further confirmation of patients with suspected lung cancer initially identified by CT image. A more comprehensive comparison is shown in Table S9 (Supporting Information). Some representative CTC‐related methods for lung cancer detection are also included. For example, a prospective cohort study in 21 French university centers reported a sensitivity of CTC detection of 26.3% and a specificity of 96.2%,^[^ ^33^ ^]^ which demonstrated that CTC enumeration for lung cancer screening is not sensitive enough. A folate‐receptor‐targeted PCR technology reported a diagnostic sensitivity of 67.2%, and a specificity of 84.1% for patients with stage I NSCLC.^[^ ^34^ ^]^ Here, we would like to highlight the potential advantages of our NSCLC CTC Score^D^ with high sensitivity and specificity. In our study, a multi‐antibody‐based CTC capture and RT‐ddPCR‐based target mRNA quantification enabled the high sensitivity and specificity of our assay for NSCLC detection. In addition, the diagnostic performance was further improved by the comprehensive model generated by machine learning.

NSCLC CTC Score^M^ exhibited a more accurate early warning as well as therapy responses than serum CYFRA21‐1 levels in monitoring the status of advanced patients, which may provide a potential blood test‐based biomarker to improve their response evaluation. The distinct molecular profiles captured by NSCLC CTC Score^D^ and NSCLC CTC Score^M^ suggest that these assays may play complementary roles in early detection and advanced disease management.

We acknowledge that the current study is limited by its sample size and that further multi‐center validation studies are needed. Moreover, comprehensive workflow integration analysis, including assays’ cost and turnaround time, is also essential to rigorously assess the overall clinical feasibility. Here, we would like to report the distinct CTC specific mRNA profiles in generating models for early NSCLC detection model and for advanced NSCLC management, and to highlight the importance of well‐defined study cohort design in clinical research.

## 4 Experimental Section

### Cell Culture

The human A549, H1299, SK‐MES‐1, and H2170 cell lines were used as models in the present study. SK‐MES‐1 and NCI‐2170 cells were purchased from Shanghai Institute of Biochemistry and Cell Biology (SIBCB) (Shanghai, China). The cell culture procedures were cultured following Cell Resource Center‐recommended culturing conditions.

### Flow Cytometry Assay

EpCAM, EGFR, and N‐cadherin on the cell surfaces were labeled by antibodies as detailed below. 2 × 10^5^ SK‐MES‐1 cells, H2170, A549, and H1299 were incubated with three antibodies (anti‐EpCAM [R&D, source‐Goat], anti‐EGFR [R&D, source‐Goat], and anti‐N‐cadherin [Proteintech, source‐Rabbit] for 30 min, respectively. Then, these samples were incubated with secondary antibodies (anti‐Goat [Abcam, source‐Donkey], anti‐Rabbit [ThemoFisher, source‐Donkey]) for 30 min, respectively. After antibody staining, the cells were washed with PBS three times, resuspended in 200 µL of PBS, and analyzed by the flow cytometer (Beckman CytoFlex S).

### Scanning Electron Microscopy (SEM)

SK‐MES‐1 cells before/after capture were fixed with 4% paraformaldehyde for 30 min at room temperature and then dehydrated with graded ethanol (30, 50, 70, 85, 95, and 100%). Cells were resuspended in 100% anhydrous ethanol and finally dropped onto a clean silicon wafer. The morphology of the cells was measured by a thermal field‐emission environmental scanning electron microscope (20.0 kV, FEI Quanta 400F) and Fe, Au elemental analysis of the samples was performed.

### Optimization of CTC Isolation Using Artificial CTC Samples

Artificial CTC Samples were prepared by spiking 200 SK‐MES‐1 cells (DiO pre‐stained) in 2 × 10^6^ PBMCs (DiI pre‐stained) isolated from benign donor's whole blood. Magnetic beads modified with different antibody concentrations were then added to the artificial samples and incubated at room temperature (RT) for 30 min.

### Gene Target Identification and Validation

Using publicly available datasets, genes for both adenocarcinomas and squamous carcinomas were selected by the following steps: i) selecting genes that were co‐expressed in at least 2 microarrays, ii) identifying absolute changes greater than 1.0‐fold with a *p*‐value of <0.05 and genes are highly expressed in NSCLC tissue and lowly expressed in normal lung tissue, iii) confirming that selected genes were lowly expressed in the immune cell populations in order to minimize background signals from non‐specifically captured leukocytes.

Prior to the clinical sample test, these genes were further validated in different samples by ddPCR: i) NSCLC cell lines, ii) peripheral blood mononuclear cells (PBMCs) freshly isolated from benign lung nodule's whole blood samples, and iii) PBMCs freshly isolated from the NSCLC samples with different stages.

### Clinical Sample Collection

Inclusion Criteria: clinical diagnosis of NSCLC or benign pulmonary nodules. a) patients with NSCLC were diagnosed by pathology/cytology, which were staged as stage I, stage II, stage III, and stage IV according to the Union for International Cancer Control criteria. b) patients with benign pulmonary nodules were confirmed by follow‐up CT images, with a minimum follow‐up period of 12 months. Exclusion Criteria: a) patients complicated with other lung diseases, b) patients with past history of lung cancer, c) patients younger than 18 years old. All patients were randomly divided into training and validation cohorts by random sampling. All procedures involving human subjects in this research were approved by the Ethics Committee of The Second Affiliated Hospital of Soochow University (JD‐LK2024021‐I01) with informed consent obtained from all participants. Peripheral venous blood was collected using a vacuum blood collection tube. To avoid the contamination from skin epithelial cells in blood draw process, the first tube during blood collection was not used in this study. CTCs isolation was completed within 24 h after the collection.

### NSCLC CTC Isolation and RNA Quantification

Multi‐antibody‐modified MNPs were prepared as follows: 0.1 mg of streptavidin‐modified beads (Beaver) were incubated for 30 min with a mixture of antibodies, including biotinylated anti‐human EpCAM (R&D Systems, polyclonal goat IgG, 200 ng/200 µL), biotinylated anti‐human EGFR (R&D Systems, polyclonal goat IgG, 200 ng/200 µL), and biotinylated anti‐human N‐cadherin (Proteintech, polyclonal rabbit IgG, 200 ng/200 µL).

Prior to CTC isolation, PBMCs from 2 mL whole blood samples were isolated using a Histopaque‐1077 density gradient (#07861, STEMCELL) in 15 mL SepMate tubes (85415, STEMCELL) with a centrifugation at 1200g, 20 min, and then resuspended in 200 µL PBS. Finally, PBMC samples were incubated with multi‐antibody‐modified MNPs for 30 min at RT to obtain purified NSCLC CTCs.

The captured NSCLC CTCs were lysed using a QIAzol (QIAGEN) lysis reagent. Direct‐zol RNA Microprep (ZYMO RESEARCH) was used to extract and purify RNA from the isolated CTCs. The collected RNA (16 µL) was reversely transcribed to complementary DNA (cDNA, 20 µL) using PrimeScript RT Master Mix (5×) (Takara) for each clinical blood sample. Six genes in three tubes, with two fluorescence filters (i.e., FAM and VIC) in each tube, were detected using 6.6 µL of cDNA. The readouts of positive and negative partitions were counted automatically by the instrument and analyzed via QX200 system (Bio‐Rad Laboratories, Inc.). All primers and probes were ordered from ThermoFisher Scientific, and detailed information is available in Table S1 (Supporting Information).

### Statical Analysis

Cell capture efficiency data were represented as mean ± standard deviation of three independent tests. NSCLC CTC score was generated by a multivariate logistic regression forward method on statistical software (SPSS v27.0). Receiver operating characteristic (ROC) curve analysis for NSCLC CTC Scores, CEA, CA199 and CA125, respectively, was performed with GraphPad Prism v10.

## Conflict of Interest

The authors declare no conflict of interest.

## Supporting information



Supporting Information

## Data Availability

The data that support the findings of this study are available from the corresponding author upon reasonable request.

## References

[1] R. L. Siegel , A. N. Giaquinto , A. Jemal , CA: Cancer J. Clin. 2024, 74, 12. 38230766 10.3322/caac.21820

[2] B. Han , R. Zheng , H. Zeng , S. Wang , K. Sun , R. Chen , L. Li , W. Wei , J. He , J. Natl. Cancer Center 2024, 4, 47. 39036382 10.1016/j.jncc.2024.01.006 PMC11256708

[3] C.T. Society, C.M. Association, C.A.A.L.C.E. Group , Chin. J. Tuberc. Respir. Dis. 2024, 47, 716.

[4] C. Henschke , R. Huber , L. Jiang , D. Yang , M. Cavic , H. Schmidt , E. Kazerooni , J. J. Zulueta , R. S. Dos Santos , L. Ventura , J. Thoracic Oncol. 2024, 19, 565. 10.1016/j.jtho.2023.11.013 37979778

[5] C.‐L. Chen , J.‐S. Hsu , Y.‐W. Shen , C.‐H. Hsu , S.‐Y. Kao , W.‐A. Lai , C.‐H. Chuang , Y.‐W. Liu , J.‐Y. Lee , S.‐H. Chou , Cancers 2024, 16, 3727.39594683

[6] P. B. Bach , J. N. Mirkin , T. K. Oliver , C. G. Azzoli , D. A. Berry , O. W. Brawley , T. Byers , G. A. Colditz , M. K. Gould , J. R. Jett , JAMA, J. Am. Med. Assoc. 2012, 307, 2418. 10.1001/jama.2012.5521 PMC3709596 22610500

[7] X. Ye , W. Fan , Z. Wang , J. Wang , H. Wang , J. Wang , C. Wang , L. Niu , Y. Fang , S. Gu , J. Cancer Res. Therapeutics 2021, 17, 1141. 10.4103/jcrt.jcrt_1485_21 34850761

[8] M. Oudkerk , S. Liu , M. A. Heuvelmans , J. E. Walter , J. K. Field , Nat. Rev. Clin. Oncol. 2021, 18, 135. 33046839 10.1038/s41571-020-00432-6

[9] M.‐L. Meyer , B. G. Fitzgerald , L. Paz‐Ares , F. Cappuzzo , P. A. Jänne , S. Peters , F. R. Hirsch , Lancet 2024, 404, 803. 39121882 10.1016/S0140-6736(24)01029-8

[10] F. Wang , X.‐Y. Diao , X. Zhang , Q. Shao , Y.‐F. Feng , X. An , H.‐Y. Wang , Cancer Commun. 2019, 39, 1. 10.1186/s40880-019-0354-z PMC6397445 30823937

[11] M. G. Krebs , R. Sloane , L. Priest , L. Lancashire , J.‐M. Hou , A. Greystoke , T. H. Ward , R. Ferraldeschi , A. Hughes , G. Clack , J. Clin. Oncol. 2011, 29, 1556. 21422424 10.1200/JCO.2010.28.7045

[12] K. Yoneda , M. Hashimoto , T. Takuwa , S. Matsumoto , Y. Okumura , S. Hasegawa , F. Tanaka , J. Clin. Oncol. 2017, 35, 23044.

[13] W. Li , J.‐B. Liu , L.‐K. Hou , F. Yu , J. Zhang , W. Wu , X.‐M. Tang , F. Sun , H.‐M. Lu , J. Deng , Mol. Cancer 2022, 21, 25. 35057806 10.1186/s12943-022-01505-z PMC8772097

[14] J. Chen , Q. Li , C.‐y. Xu , Y.‐y. Qian , Z.‐h. Yang , D.‐s. Chen , J. Clin. Oncol. 2022, 40, 21018.

[15] T. H. Kim , Y. Wang , C. R. Oliver , D. H. Thamm , L. Cooling , C. Paoletti , K. J. Smith , S. Nagrath , D. F. Hayes , Nat. Commun. 2019, 10, 1478. 30932020 10.1038/s41467-019-09439-9 PMC6443676

[16] M. Lim , C.‐J. Kim , V. Sunkara , M.‐H. Kim , Y.‐K. Cho , Micromachines 2018, 9, 100. 30424034 10.3390/mi9030100 PMC6187707

[17] S. H. Hussain , C. S. Huertas , A. Mitchell , A.‐L. Deman , E. Laurenceau , Biosens. Bioelectronics 2022, 197, 113770. 10.1016/j.bios.2021.113770 34768065

[18] L.‐N. Qi , B.‐D. Xiang , F.‐X. Wu , J.‐Z. Ye , J.‐H. Zhong , Y.‐Y. Wang , Y.‐Y. Chen , Z.‐S. Chen , L. Ma , J. Chen , Cancer Res. 2018, 78, 4731. 29915159 10.1158/0008-5472.CAN-17-2459

[19] M. Ciccioli , N. Bravo‐Santano , A. Davis , J. Lewis , R. Malcolm , A.‐S. Pailhes‐Jimenez , Cancer Res. 2021, 81, 588.

[20] M. Zeinali , M. Lee , A. Nadhan , A. Mathur , C. Hedman , E. Lin , R. Harouaka , M. S. Wicha , L. Zhao , N. Palanisamy , Cancers 2020, 12, 127. 31947893 10.3390/cancers12010127 PMC7016759

[21] R. G. Austin , T. J. Huang , M. Wu , A. J. Armstrong , T. Zhang , Adv. Drug Delivery Rev. 2018, 125, 132. 10.1016/j.addr.2018.01.013 29366804

[22] S. Ju , C. Chen , J. Zhang , L. Xu , X. Zhang , Z. Li , Y. Chen , J. Zhou , F. Ji , L. Wang , Biomarker Res. 2022, 10, 58. 10.1186/s40364-022-00403-2 PMC9375360 35962400

[23] G.‐C. Duan , X.‐P. Zhang , H.‐E. Wang , Z.‐K. Wang , H. Zhang , L. Yu , W.‐F. Xue , Z.‐F. Xin , Z.‐H. Hu , Q.‐T. Zhao , OncoTargets Therapy 2020, 13, 1931. 32184628 10.2147/OTT.S241956 PMC7061431

[24] D. Han , X.‐H. Ren , X.‐R. Liao , X.‐Y. He , T. Guo , X.‐S. Chen , X. Pang , S.‐X. Cheng , Nano Lett. 2023, 23, 3678. 37052638 10.1021/acs.nanolett.2c04643

[25] J. Ma , Y. Chen , J. Ren , T. Zhou , Z. Wang , C. Li , L. Qiu , T. Gao , P. Ding , Z. Ding , ACS Sens. 2023, 8, 3744. 37773014 10.1021/acssensors.3c01063

[26] H. Xu , Y. Zuo , S. Gao , Y. Liu , T. Liu , S. He , M. Wang , L. Hu , C. Li , Y. Yu , Small 2024, 20, 2310360. 10.1002/smll.202310360 38698606

[27] E. Purcell , Z. Niu , S. Owen , M. Grzesik , A. Radomski , A. Kaehr , N.‐E. Onukwugha , H. F. Winkler , N. Ramnath , T. Lawrence , Cell Rep. 2024, 43, 113687. 38261515 10.1016/j.celrep.2024.113687

[28] Z. Dong , Y. Wang , G. Xu , B. Liu , Y. Wang , J. Reboud , P. Jajesniak , S. Yan , P. Ma , F. Liu , Proc. Natl. Acad. Sci. USA 2024, 121, 2315168121. 10.1073/pnas.2315168121 PMC11087790 38683997

[29] P. Saintigny , S. Coulon , M. Kambouchner , S. Ricci , E. Martinot , C. Danel , J. L. Breau , J. F. Bernaudin , Int. J. Cancer 2005, 115, 777. 15729695 10.1002/ijc.20942

[30] O. Nordgård , G. Singh , S. Solberg , L. Jørgensen , A. R. Halvorsen , R. Smaaland , O. T. Brustugun , Å. Helland , PLoS One 2013, 8, 62153. 10.1371/journal.pone.0062153 PMC3643953 23671585

[31] A. M. Newman , S. V. Bratman , J. To , J. F. Wynne , N. C. Eclov , L. A. Modlin , C. L. Liu , J. W. Neal , H. A. Wakelee , R. E. Merritt , Nat. Med. 2014, 20, 548. 24705333 10.1038/nm.3519 PMC4016134

[32] Q. Du , R. Yu , H. Wang , D. Yan , Q. Yuan , Y. Ma , D. Slamon , D. Hou , H. Wang , Q. Wang , Clin. Respiratory J. 2018, 12, 2020. 10.1111/crj.12769 29356386

[33] C.‐H. Marquette , J. Boutros , J. Benzaquen , M. Ferreira , J. Pastre , C. Pison , B. Padovani , F. Bettayeb , V. Fallet , N. Guibert , Lancet Respiratory Med. 2020, 8, 709. 10.1016/S2213-2600(20)30081-3 32649919

[34] Y. Yu , Z. Chen , J. Dong , P. Wei , R. Hu , C. Zhou , N. Sun , M. Luo , W. Yang , R. Yao , Translational Oncol. 2013, 6, 697. 10.1593/tlo.13535 PMC3890704 24466372

