# Supplementary material for: Distinct CTC Specific RNA Profile Enables NSCLC Early Detection and Dynamic Monitoring of Advanced NSCLC
Source: Adv Sci (Weinh). 2025 Apr 3;12(23):2417849. doi: 10.1002/advs.202417849 (PMC12199372; doi:10.1002/advs.202417849)
Supplement: Supplementary file 1 — Supporting Information [file ADVS-12-2417849-s001.docx]

Supporting Information

**Distinct CTC Specific RNA Profiles Enables NSCLC Early Detection and Dynamic Monitoring of Advanced NSCLC**

Xiaoyu Wang^a,b,#^, Pi Ding^a,#^, Wenjuan Xu^a,b^, Lei Qiu^b,c^, Jing Ren^b,d^, Yucheng Fei^b,c^, Zhili Wang^b^, Cheng Li^b,c^, Yufei Xing^a^, Mingjing Shen^e^, Yawen Zhu^a^, Yun Guo^a^, Na Sun^b,c,^*, Renjun Pei^b,c,^*, Minhua Shi^a,^*

^a^ Department of Pulmonary and Critical Care Medicine, The Second Affiliated Hospital of Soochow University, Suzhou, 215004, China

^b^ CAS Key Laboratory for Nano-Bio Interface, Suzhou Institute of Nano-Tech and Nano-Bionics, Chinese, Academy of Sciences, Suzhou 215123, China

^c^ School of Nano-Tech and Nano-Bionics, University of Science and Technology of China, Hefei 230026, China

^d^ Department of Gynecology and Obstetrics, The Second Affiliated Hospital of Soochow University, Suzhou, 215004, China

^e^ Thoracic Department, The Second Affiliated Hospital of Soochow University, Suzhou, 215004, China

^#^ Xiaoyu Wang and Pi Ding contributed equally.

*** Corresponding authors.**

**Email:** [shiminhua@163.com](mailto:shiminhua@163.com); [nsun2013@sinano.ac.cn](mailto:nsun2013@sinano.ac.cn); [rjpei2011@sinano.ac.cn](mailto:rjpei2011@sinano.ac.cn)

**SUPPLEMENTARY ASSESSMENT CRITERIA**

**The RECIST 1.1 assessment criteria.**

**SUPPLEMENTARY FIGURES**

**Supplementary Figure S1.** Expression heatmap of the 6-gene panel in NSCLC tissues and normal lung tissues.

**Supplementary Figure S2.** 6 genes corresponding to pathway analysis, molecular function, cellular component and biological process analysis.

**Supplementary Figure S3.** SEM images of SK-MES-1 cell before and after purification by multi-antibody-modified MNPs.

**Supplementary Figure S4.** The expression levels of EpCAM, EGFR, and N-cadherin on these four cell lines were analyzed by flow cytometry.

**Supplementary Figure S5** Validation of reproducibility of CTC RNA assay using clinical samples.

**Supplementary Figure S6.** Confusion matrix analysis of (A) CTC ScoresD and (B) CTC Scores after LOOCV validation in differentiating early-stage NSCLC from benign controls.

**Supplementary Figure S7.** A) Receiver operating characteristic curve analysis and B-E) confusion matrix analysis of CTC Scores^D^ and serum markers in differentiating all-stage NSCLC from benign controls.

**Supplementary Figure S8.** A) Receiver operating characteristic curve analysis and B-E) confusion matrix analysis of CTC Scores^D^ and serum markers in differentiating early-stage NSCLC from benign controls.

**Supplementary Figure S9.** Boxplot of NSCLC CTC-derived six mRNA expression levels of individuals in different types of advanced NSCLC samples (adenocarcinoma (n=22) and squamous carcinoma (n=8)).

**SUPPLEMENTARY TABLE**

**Supplementary Table 1.** Primers and probes for NSCLC CTC RNA assay.

**Supplementary Table 2.** Clinical characteristics of the early-stage NSCLC cohort.

**Supplementary Table 3.** Clinical characteristics of the benign donor cohort.

**Supplementary Table 4.** Clinical characteristics of the advanced NSCLC cohort.

**Supplementary Table 5.** Other cancers cohort of test by NSCLC Score^D^.

**Supplementary Table 6.** The area under the NSCLC CTC Score^D^ ROC curve of the 6-gene panel.

**Supplementary Table 7.** Comparison of diagnostic capacity in training and test cohort under different methods.

**Supplementary Table 8.** The area under the NSCLC CTC Score^M^ ROC curve of the 6-gene panel.

**Supplementary Table 9.** Comparison of our developed CTC RNA assay with current clinical testing technologies and other novel liquid biopsy methods.

**SUPPLEMENTARY ASSESSMENT CRITERIA**

**The RECIST 1.1 assessment criteria**

RECIST 1.1 (Response Evaluation Criteria in Solid Tumors version 1.1) is a standardized criterion used to assess the treatment response of solid tumors, primarily through imaging examinations (such as CT or MRI) to determine changes in tumor size. According to the RECIST 1.1 standards, tumor response is categorized into four classes: Complete Response (CR, where all target lesions disappear), Partial Response (PR, where the total sum of diameters of target lesions decreases by at least 30%), Stable Disease (SD, where the changes in target lesions do not meet the criteria for either PR or Progressive Disease), and Progressive Disease (PD, where the target lesions increase by 20% or new lesions appear).

**SUPPLEMENTARY FIGURE**

**Figure S1**


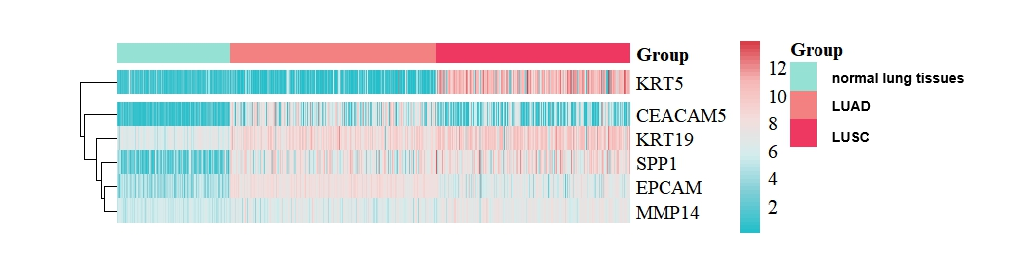
**Figure S1.** Expression heatmap of the 6-gene panel in NSCLC tissues and normal lung tissues.

**Figure S2**


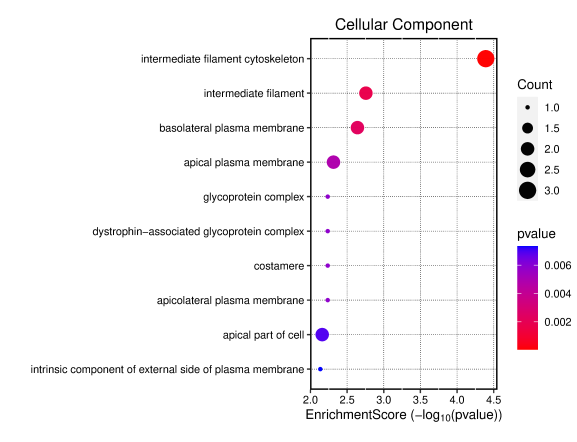

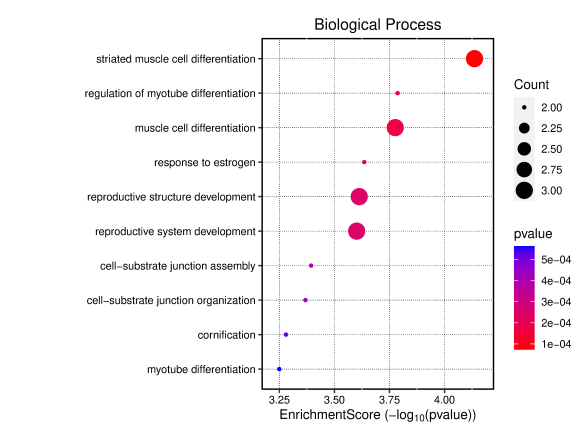

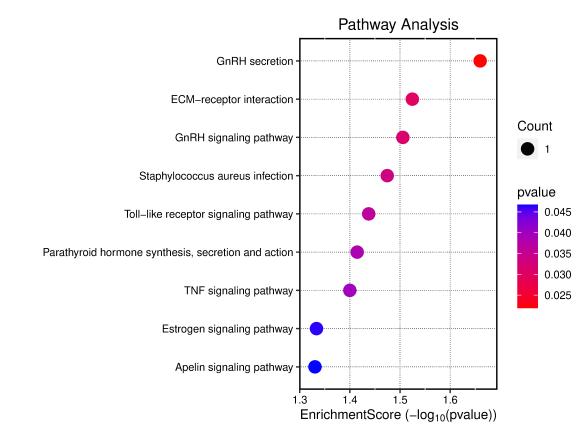

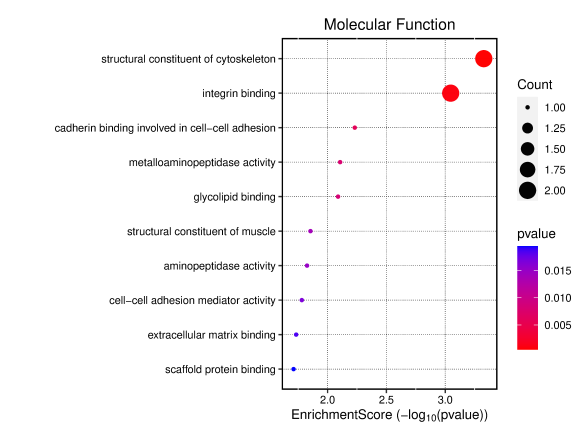


**A**

**B**

**C**

**D**

**Figure S2.** 6 genes corresponding to pathway analysis, molecular function, cellular component and biological process analysis.

**Figure S3**

**
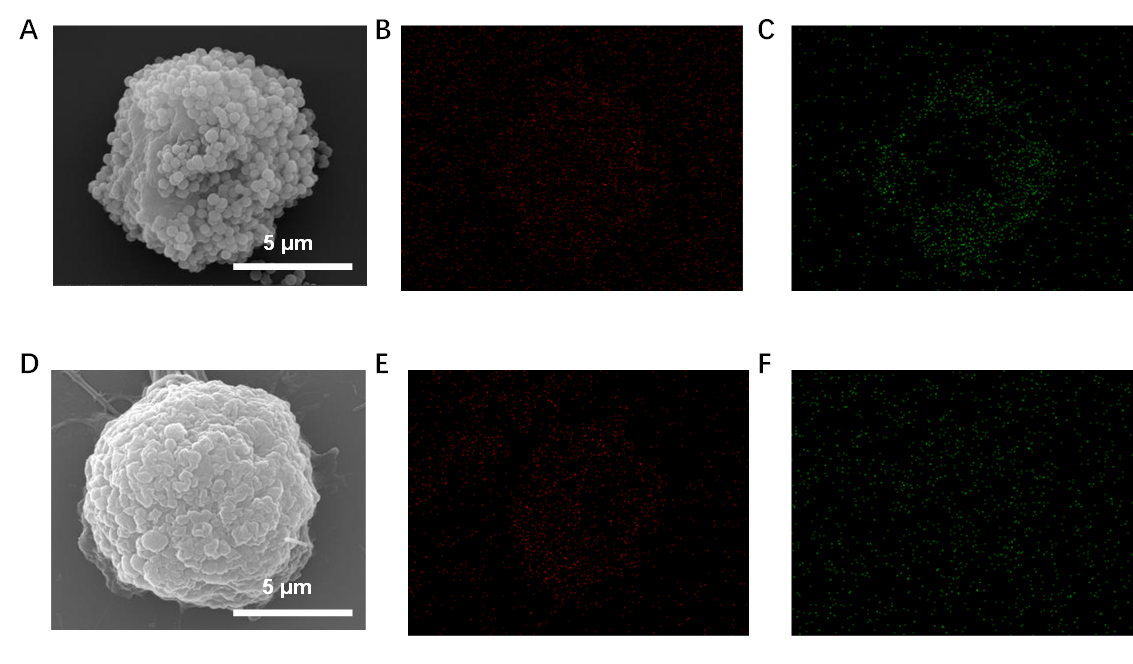
 Figure S3.** SEM images of SK-MES-1 cell before and after purification by multi-antibody-modified MNPs. (A-C) captured SK-MES-1 cell by multivalent antibody-modified MNPs and the analysis of Au, Fe distribution. (D-F) cell before captured by multivalent antibody-modified MNPs and the analysis of Au, Fe distribution.

**Figure S4**


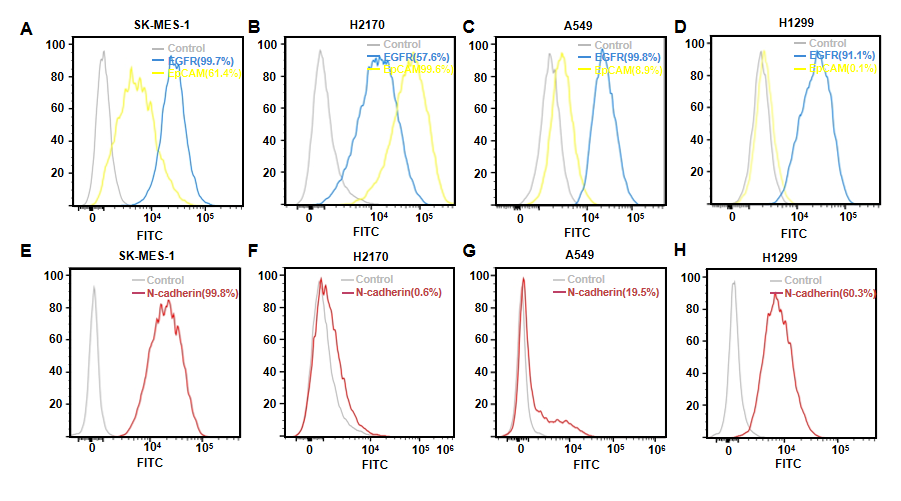


**Figure S4.** The expression levels of EpCAM, EGFR, and N-cadherin on these four cell lines were analyzed by flow cytometry.

**Figure S5**

**
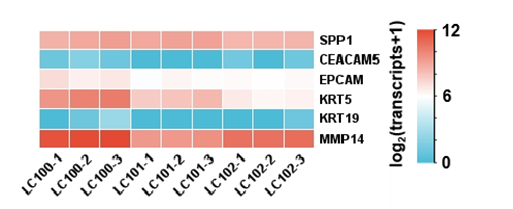
**

**Figure S5.** Validation of reproducibility of CTC RNA assay using clinical samples. Heatmap of 6-gene expression levels in triplicate tests for three NSCLC stage I-II patients.

**Figure S6**


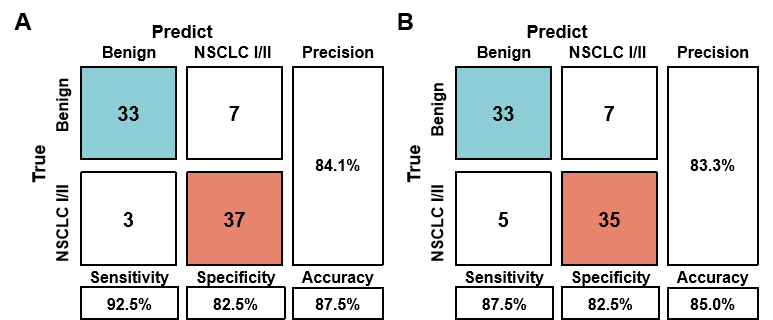


**Figure S6.** Confusion matrix analysis of (A) CTC Scores^D^ and (B) CTC Scores after LOOCV validation in differentiating early-stage NSCLC from benign controls.

**Figure S7**


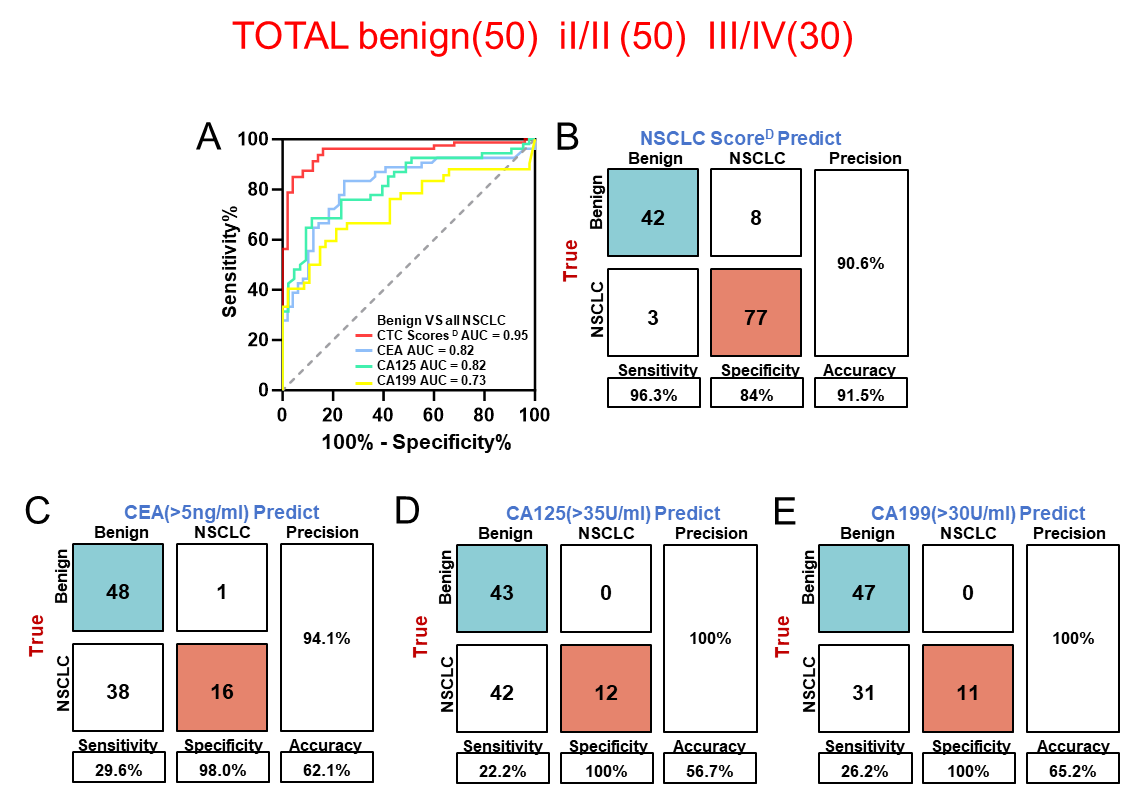


**Figure S7.** A) Receiver operating characteristic curve analysis and B-E) confusion matrix analysis of CTC Scores^D^ and serum markers in differentiating all-stage NSCLC from benign controls.

**Figure S8**

**
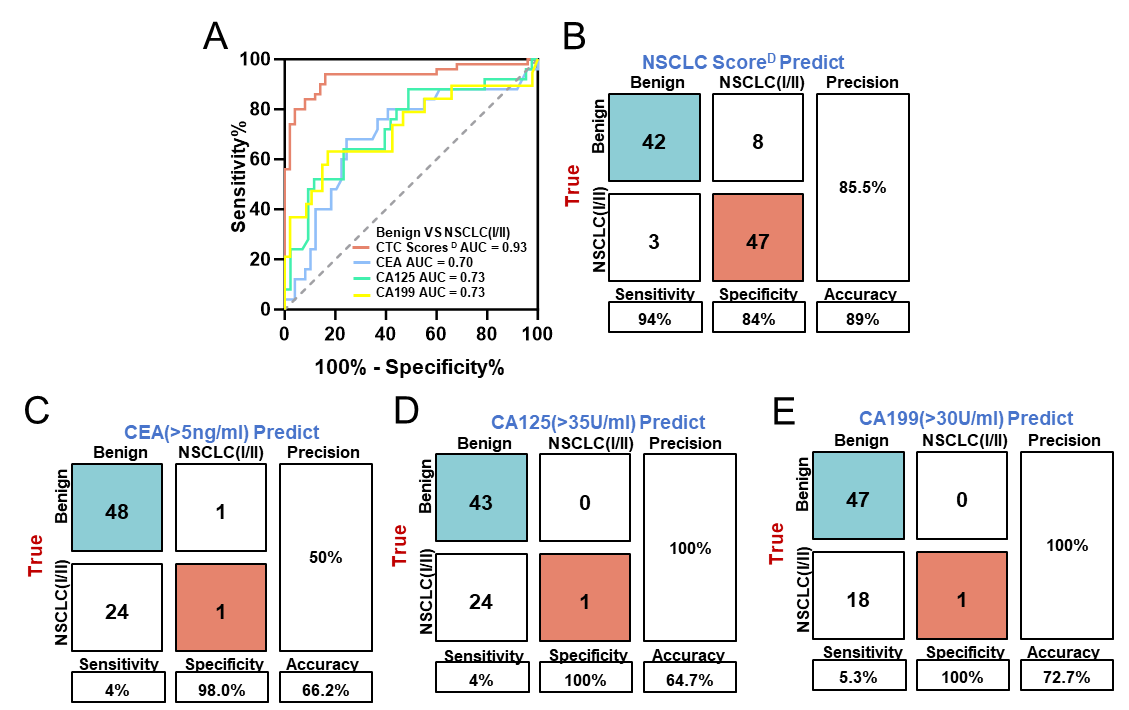
**

**Figure S8. A)** Receiver operating characteristic curve analysis and **B-E**) confusion matrix analysis of CTC Scores^D^ and serum markers in differentiating early-stage NSCLC from benign controls.

**Figure S9**

**
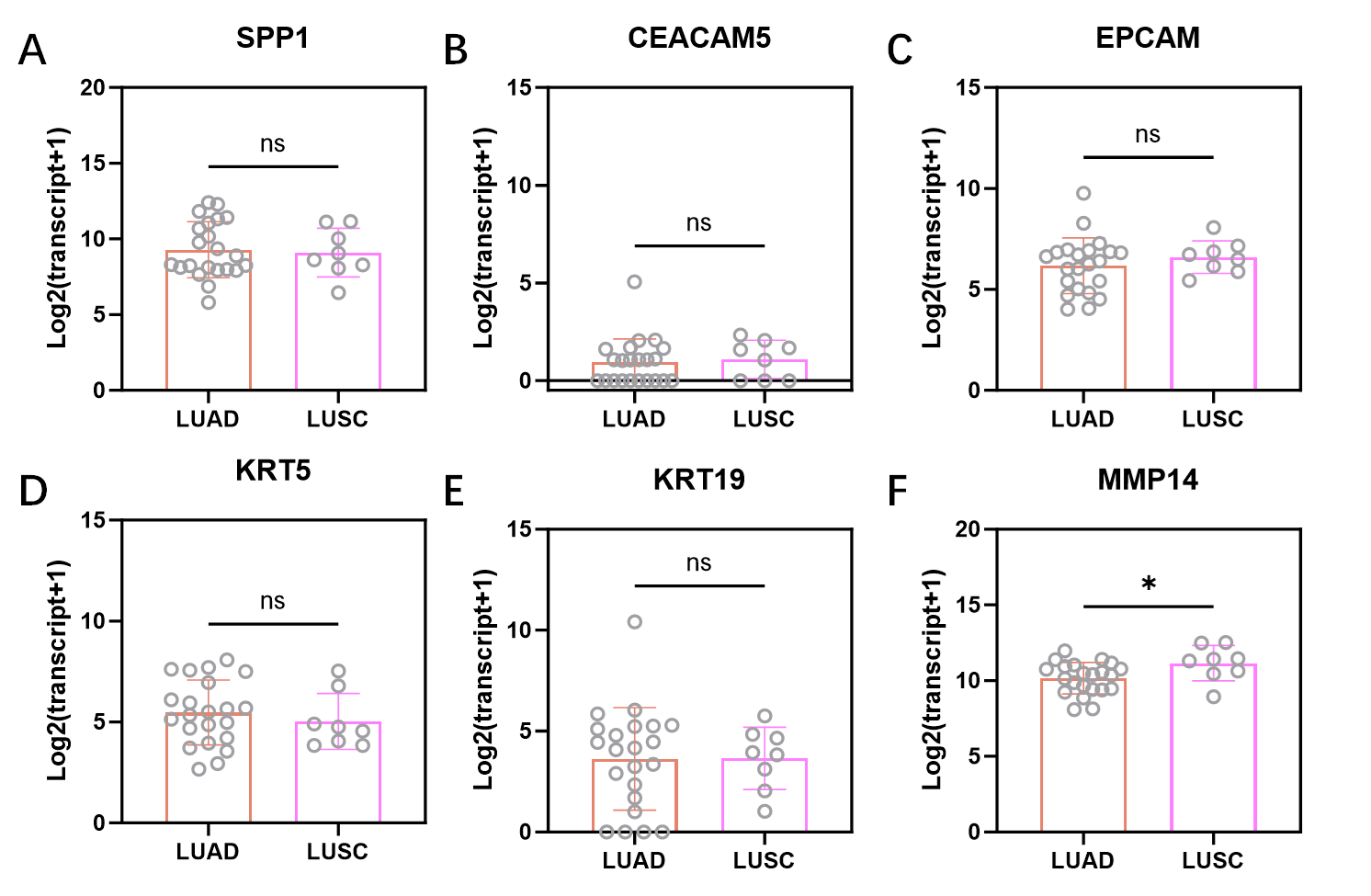
**

**Figure S9.** (A-F) boxplot of NSCLC CTC-derived six mRNA expression levels of individuals in different types of advanced NSCLC samples (adenocarcinoma(n=22) and squamous carcinoma(n=8)).

**SUPPLEMENTARY TABLE**

**Table S1.** Primers and probes for NSCLC CTC RNA assay.

| Gene | Thermo Fisher Scientific Assay ID |
| --- | --- |
| *SPP1* | [Hs00959010_m1](https://www.thermofisher.cn/taqman-gene-expression/product/Hs00959010_m1?CID=&ICID=&subtype=) |
| *CEACAM5* | [Hs00944025_m1](https://www.thermofisher.cn/taqman-gene-expression/product/Hs00944025_m1?CID=&ICID=&subtype=) |
| *EPCAM* | Hs00901885 m1 |
| *KRT5* | Hs00361185 m1 |
| *KRT19* | Hs01051611 gH |
| *MMP14* | [Hs00237119_m1](https://www.thermofisher.cn/taqman-gene-expression/product/Hs00237119_m1?CID=&ICID=&subtype=) |

**Table S2.** Clinical characteristics of the early-stage NSCLC cohort.

| **Characteristics** | **NSCLC(I/II)(N=50)** |
| --- | --- |
| **Gender, n [%]** |  |
| female | 17[34%] |
| male | 33[66%] |
| **Age** |  |
| Mean ± SD | 63 ± 12 |
| Median [min-max] | 64 [32, 87] |
| **Smoking status,n [%]** |  |
| Smokers | 4[8%] |
| Previous | 3[6%] |
| Non smokers | 43[86%] |
| **Histological type,n [%]** |  |
| Adenocarcinoma | 50[100%] |
| Squamous cell | 0[0%] |
| **Pathologic stage,n [%]** |  |
| IA |  |
| TisN0M0 | 8[16%] |
| T1aN0M0 | 4[8%] |
| T1bN0M0 | 20[40%] |
| T1cN0M0 | 10[20%] |
| IB |  |
| T2aN0M0 | 5[10%] |
| IIA | 1[2%] |
| IIB | 2[4%] |
| **CEA** |  |
| Mean ± SD | 2.5 ± 3.4 |
| Median [min-max] | 1.78 [0.3, 18.6] |
| **CA125** |  |
| Mean ± SD | 13.8 ± 9.4 |
| Median [min-max] | 11.30 [3.81, 45.9] |
| **CA199** |  |
| Mean ± SD | 14.9± 9.84 |
| Median [min-max] | 12 [2.0, 30.9] |

**Table S3.** Clinical characteristics of the benign donor cohort.

| **Characteristics** | **Benign donors (N=50)** |
| --- | --- |
| **Gender, n [%]** |  |
| female | 15[30%] |
| male | 35[70%] |
| **Age** |  |
| Mean ± SD | 50 ± 7 |
| Median [min-max] | 52 [32, 69] |
| **CEA** |  |
| Mean ± SD | 0.97 ± 0.5 |
| Median [min-max] | 1.0[0.46, 5.37] |
| **CA125** |  |
| Mean ± SD | 8.6 ± 3.8 |
| Median [min-max] | 7.6 [3.0, 25.7] |
| **CA199** |  |
| Mean ± SD | 7.4 ± 5.0 |
| Median [min-max] | 5.6 [3.4, 24.5] |

**Table S4.** Clinical characteristics of the advanced NSCLC cohort.

| **Characteristics** | **advanced NSCLC(III/IV)(N=30)** |
| --- | --- |
| **Gender, n [%]** |  |
| female | 9[30%] |
| male | 21[70%] |
| **Age** |  |
| Mean ± SD | 69 ± 11 |
| Median [min-max] | 70 [32, 87] |
| **Smoking status,n [%]** |  |
| Smokers | 14[46.7%] |
| Previous | 6[20%] |
| Non smokers | 10[33.3%] |
| **Histological type,n [%]** |  |
| Adenocarcinoma | 22[73.3] |
| Squamous cell | 8[26.7%] |
| **CEA** |  |
| Mean ± SD | 34.8 ± 94.5 |
| Median [min-max] | 5.1[0.3, 507] |
| **CA125** |  |
| Mean ± SD | 62.3 ± 86.7 |
| Median [min-max] | 26.8 [5.44, 447.0] |
| **CA199** |  |
| Mean ± SD | 66.7 ± 118.4 |
| Median [min-max] | 13.3 [2.0, 438.0] |

**Table S5.** Other cancers cohort of test by NSCLC Score^D^.

| Sample ID | Gender | Age | Histological type | Pathologic stage |
| --- | --- | --- | --- | --- |
| HCC1 | female | 57 | Hepatocellular cancer | II |
| HCC8 | female | 65 | Hepatocellular cancer | IB |
| HCC11 | male | 71 | Hepatocellular cancer | IB |
| HCC17 | male | 48 | Hepatocellular cancer | II |
| HCC18 | male | 67 | Hepatocellular cancer | IB |
| CRC145 | female | 57 | colorectal cancer | IIA |
| CRC174 | female | 71 | colorectal cancer | I |
| CRC181 | male | 63 | colorectal cancer | IIA |
| CRC213 | male | 64 | colorectal cancer | IIA |
| BC17 | female | 53 | breast cancer | IB |
| BC24 | female | 52 | breast cancer | II |
| BC29 | female | 68 | breast cancer | IB |

NSCLC, Non-Small Cell Lung Cancer; HCC, hepatocellular cancer; BC, breast cancer; CA125, carbohydrate antigen 125; CEA, carcinoembryonic antigen; CA199, carbohydrate antigen199.

**Table S6.** The area under the NSCLC CTC Score^D^ ROC curve of the 6-gene panel.

| Gene | Area | Std.Error | 95%CI | P value |
| --- | --- | --- | --- | --- |
| *SPP1* | 0.8688 | 0.03998 | 0.7904 to 0.9471 | <0.0001 |
| *CEACAM5* | 0.6313 | 0.06262 | 0.5085 to 0.7540 | 0.0433 |
| *EPCAM* | 0.8606 | 0.04248 | 0.7774 to 0.9439 | <0.0001 |
| *KRT5* | 0.5363 | 0.06904 | 0.4009 to 0.6716 | 0.5768 |
| *KRT19* | 0.6406 | 0.06279 | 0.5176 to 0.7637 | 0.0304 |
| *MMP14* | 0.7950 | 0.04909 | 0.6988 to 0.8912 | <0.0001 |

ROC, receiver operating characteristic; CI, confidence interval

**Table S7.** Comparison of diagnostic capacity in training and test cohort under different methods.

| **NSCLC(I/II) vs Benign** | | | | | | | | |
| --- | --- | --- | --- | --- | --- | --- | --- | --- |
| **Training Cohort** | | | | | | | | |
| **Methods** | **ROC curve** | | | **Diagnostic test** | | | | |
|  | **AUC** | **p-value** | **cut-off** | **SEN(%)** | **SPE(%)** | **PPV(%)** | **NPV(%)** | **AI(%)** |
| NSCLC CTC Score^D^ | 0.913 | <0.0001 | -0.4554 | 92.5 | 82.5 | 84.1 | 91.7 | 87.5 |
| CEA | 0.665 | 0.043 | 5 | 0 | 97.5 | 0 | 67.2 | 66.1 |
| CA125 | 0.726 | 0.006 | 35 | 0 | 100 | 0 | 66.1 | 66.1 |
| CA199 | 0.722 | 0.010 | 30 | 6.25 | 100 | 100 | 72.2 | 72.7 |
| **Independent Test Cohort** | | | | | | | | |
| **Methods** | **ROC curve** | | | **Diagnostic test** | | | | |
|  | **AUC** | **p-value** | **cut-off** | **SEN(%)** | **SPE(%)** | **PPV(%)** | **NPV(%)** | **AI(%)** |
| NSCLC CTC Score^D^ | 1.000 | 0.0002 | -0.4554 | 100 | 90 | 90.9 | 100 | 95 |
| CEA | 0.796 | 0.059 | 5 | 16.7 | 100 | 100 | 64.3 | 66.7 |
| CA125 | 0.611 | 0.522 | 35 | 16.7 | 100 | 100 | 54.5 | 58.3 |
| CA199 | 0.958 | 0.025 | 30 | 0 | 100 | 0 | 0 | 72.7 |

ROC, receiver operating characteristic; SEN, sensitivity; SPE, specificity; PPV, positive predictive value; NPV, negative predictive value; AI, accuracy index;

**Table S8.** The area under the NSCLC CTC Score^M^ ROC curve of the 6-gene panel.

| Gene | Area | Std.Error | 95%CI | P value |
| --- | --- | --- | --- | --- |
| *SPP1* | 0.9517 | 0.02314 | 0.9063 to 0.9970 | <0.0001 |
| *CEACAM5* | 0.7167 | 0.06551 | 0.5883 to 0.8451 | 0.0020 |
| *EPCAM* | 0.9617 | 0.01909 | 0.9242 to 0.9991 | <0.0001 |
| *KRT5* | 0.5558 | 0.07407 | 0.4107 to 0.7010 | 0.4265 |
| *KRT19* | 0.8400 | 0.05508 | 0.7320 to 0.9480 | <0.0001 |
| *MMP14* | 0.8883 | 0.03907 | 0.8118 to 0.9649 | <0.0001 |

ROC, receiver operating characteristic; CI, confidence interval

**Table S9.** Comparison of our developed CTC RNA assay with current clinical testing technologies and other novel liquid biopsy methods.

| **Study type** | **biomarker type** | **samples** | **time** | **Cost ($)** | **Sensitivity**  **(%)** | **Specificity (%)** | **AUC** | **Ref** |
| --- | --- | --- | --- | --- | --- | --- | --- | --- |
| **Our study** | CTC scores^D^ | 50 stage I II NSCLC | <4h | 100 | 94 | 84 | 0.93 |  |
| **Standard clinical techniques** | LD-CT | 288 Lung cancer | 2h | 200 | 93.8 | 73.4 |  | ^[1]^ |
|  | ProGRP | 248 Lung cancer | <4h | 69 | 28.63 | 72.88 | 0.587 | ^[2]^ |
|  | CEA |  |  |  | 42.34 | 93.22 | 0.792 |  |
|  | CYFRA 21-1 |  |  |  | 63.71 | 71.19 | 0.792 |  |
|  | CA724 |  |  |  | 22.18 | 91.53 | 0.663 |  |
|  | SCC |  |  |  | 34.68 | 76.27 | 0.562 |  |
|  | NSE |  |  |  | 41.94 | 76.27 | 0.581 |  |
|  | ProGRP + CEA + NSE + CYFRA 21-1 + CA72-4 + SCC |  |  |  | 90.73 | 33.90 | - |  |
|  | Seven TAAbs | 175 stage I II NSCLC | 24-48h | 110 | 56.57 | 91.6 | - | ^[3]^ |
| **ctDNA-based technologies** | DNA Methylation | 284 Lung cancer | - | - | 81 | 91.4 | 0.89 | ^[4]^ |
|  | ctDNA | 414 stage I LUAD | - | - | 31.2 | 90 | - | ^[5]^ |
| **CTC-related methods** | enumeration | 614 Lung cancer | - | - | 26.3 | 96.2 | - | ^[6]^ |
|  | FR-based PCR | 67 stage I NSCLC | - | - | 67.2 | 84.1 | - | ^[7]^ |

**References**

[1] N. L. S. T. R. Team, *New England Journal of Medicine* 2013, *368* (21), 1980.

[2] H. Tong, B. Dan, H. Dai, M. Zhu, *Future Oncology* 2022, *18* (11), 1357.

[3] Q. Du, R. Yu, H. Wang, D. Yan, Q. Yuan, Y. Ma, D. Slamon, D. Hou, H. Wang, Q. Wang, *The Clinical Respiratory Journal* 2018, *12* (6), 2020.

[4] C. Zhang, W. Yu, L. Wang, M. Zhao, Q. Guo, S. Lv, X. Hu, J. Lou, *Journal of Cancer* 2017, *8* (17), 3585.

[5] T. H. Hong, S. Hwang, A. Dasgupta, C. Abbosh, T. Hung, J. Bredno, J. Walker, X. Shi, T. Milenkova, L. Horn, *Journal of Thoracic Oncology* 2024, *19* (11), 1512.

[6] C.-H. Marquette, J. Boutros, J. Benzaquen, M. Ferreira, J. Pastre, C. Pison, B. Padovani, F. Bettayeb, V. Fallet, N. Guibert, *The Lancet Respiratory Medicine* 2020, *8* (7), 709.

[7] Y. Yu, Z. Chen, J. Dong, P. Wei, R. Hu, C. Zhou, N. Sun, M. Luo, W. Yang, R. Yao, *Translational oncology* 2013, *6* (6), 697.
